# Probiotic-Fermented Distillers Grain Alters the Rumen Microbiome, Metabolome, and Enzyme Activity, Enhancing the Immune Status of Finishing Cattle

**DOI:** 10.3390/ani13243774

**Published:** 2023-12-07

**Authors:** Shihui Mei, Guangxia He, Ze Chen, Rong Zhang, Yixiao Liao, Mingming Zhu, Duhan Xu, Yanjuan Shen, Bijun Zhou, Kaigong Wang, Chunmei Wang, Erpeng Zhu, Chao Chen

**Affiliations:** College of Animal Science, Guizhou University, Guiyang 550025, China; shihuimei0@163.com (S.M.); guangxiahe0@163.com (G.H.); 14785135914@163.com (Z.C.); zr15185362945@163.com (R.Z.); 18684164223@163.com (Y.L.); 18608518394@163.com (M.Z.); l8718227x1@163.com (D.X.); 18275454821@163.com (Y.S.); bjzhou@gzu.edu.cn (B.Z.); as.kgwang@gzu.edu.cn (K.W.); cmwang3@gzu.edu.cn (C.W.); zhu13782701756@126.com (E.Z.)

**Keywords:** probiotic-fermented distillers grain, antioxidant capacity, immune status, microbiome, metabolome

## Abstract

**Simple Summary:**

Feedstuff deficiency has forced researchers to find new natural alternatives. Distillers grain (DG) is a high-quality feed alternative due to its rich nutrients. However, the use of DG is limited in livestock diets due to the presence of some antinutritional compounds. The probiotic fermentation of DG can improve its nutritional composition, eliminating the antinutrient composition of DG. In this study, feeding with 15% probiotic-fermented distillers grains (PFDG) improved the antioxidant capacity, immune status, and rumen enzyme activity, without affecting the ADG, of finishing cattle, regulating rumen microbiota abundance and showing a positive effect on rumen phenylalanine metabolism, tryptophan metabolism, and pyrimidine metabolism. The current results indicate that PFDG could be incorporated, at a 15% inclusion rate, into the diet in finishing cattle.

**Abstract:**

A total of 30 Simmental crossbred cattle (6.50 months old, 265.0 ± 22.48 kg) were randomly divided into three groups, with 10 heads per group, and fed for 45 days. The diet treatments consisted of the Control group without PFDG supplementation, the PFDG-15% group with 15% PFDG substituting for 15% concentrate, and PFDG-30% group with 30% PFDG substituting for 30% concentrate. The results showed that compared with the Control group, the average daily gain (ADG) of the cattle in the PFDG-30% group decreased significantly (0.890 vs. 0.768 kg/d, *p* = 0.005). The serum malondialdehyde content of cattle in the PFDG-15% and PFDG-30% groups decreased significantly (*p* = 0.047) compared to that of the Control group. However, the serum superoxide dismutase activity of cattle in the PFDG-30% group was significantly higher than that of the Control group (*p* = 0.047). Meanwhile, both the PFDG-15% and PFDG-30% groups (1758.47 vs. 2061.30 μg/mL) showed higher serum levels of immunoglobulin G, while the interleukin-10 concentration was lower in the PFDG-30% group (*p* = 0.027). In addition, the PFDG-15% and PFDG-30% groups shifted the rumen microbiota by improving the abundances of *F082* (related to propionic acid production) and fiber-degrading bacteria (*Lachnospiraceae_UGG-009* and *Prevotellaceae_UCG-001*) and reducing the abundance of the disease-associated bacteria *Selenomonas*. A Kyoto encyclopedia of genes and genomes (KEGG) analysis illustrated that three key metabolic pathways, including phenylalanine metabolism, pyrimidine metabolism, and tryptophan metabolism, were enriched in the PFDG-15% group, but eight key metabolic pathways, including arachidonic acid metabolism, were enriched in the PFDG-30% group. Importantly, both the PFDG-15% and PFDG-30% groups increased (*p* < 0.01) the activities of cellulase, lipase, and protease in the rumen. Finally, the different bacterial abundance in the rumen was associated with changes in the ADG, serum antioxidant capacity, immune status, rumen enzyme activity, and metabolites. These results suggest that PFDG alters rumen microbiome abundance, metabolome, and enzyme activity for enhancing serum antioxidant capacity and the immune status, but when the supplemental level reaches 30%, it has a negative effect on ADG and the anti-inflammatory factors in finishing cattle.

## 1. Introduction

Currently, with the increase in the population and the vigorous development of the breeding industry, the problem of feedstuff deficiency, which is the greatest constraint on livestock development in China, is becoming more and more prominent [1]. Soybeans and other grains, as the current major sources of protein feed, may be viewed as a direct competition with human food security [2]. In addition, agricultural land currently used for livestock feed production is under pressure for its conversion into crop land [3]. Therefore, natural feed alternatives need to be found to replace the current supply and to meet the growing needs of animals.

Distillers grains (DG) are by-products from the process of brewing grain crops, including sorghum, wheat, corn, and rye, and about 100 million tons of DG are produced annually in China [4]. DG is rich in protein, fiber, and fat, and is widely used as a high-quality feed alternative in livestock diets due to its nutritious and productive advantages [5,6].

However, since DG is prone to mycotoxin production due to improper management, direct utilization will lead to an increasing risk of poisoning in livestock [7]. Drying treatment raises the price of DG, and excessive heating during drying also renders it more susceptible to protein damage and reduced amino acid availability [8]. In contrast, fermentation treatment and the use of mixed silage can better extend the storage time and improve the quality of DG, to a certain extent [9]. In particular, Probiotic-fermented DG (PFDG) has the advantage of improving the quality and palatability of DG by playing a probiotic role, and it has become an attractive focus of research in the field of DG re-utilization [10].

PFDG is rich in probiotics and probiotic metabolites; probiotics can regulate the redox status of animals through their own antioxidant system [11], and the active polyphenol compounds rich in DG also exhibit strong antioxidant activities [12]. In addition, the organic acids and bacteriocins produced by probiotics can inhibit the proliferation of harmful bacteria, including salmonella and Escherichia coli, maintain the gastrointestinal microbiota balance and the health of livestock, and improve animal immunity [13,14]. As an important digestive organ for ruminants, rumen contains rich microflora, which can also produce various metabolites [15]. Changes in the rumen microbiota and metabolites can be used to assess the physiological or pathological status of an organism [16,17]. However, at present, feeding with PFDG mainly focuses on improving animal growth performance and meat quality [18,19], and there are few reports on the effects of a PFDG diet on the immune status, antioxidant indexes, rumen enzyme activity, microbiome, and metabolome of cattle.

Therefore, here, we used the four probiotics (*Lactobacillus plantarum*, *Aspergillus Niger*, *Saccharomyces cerevisiae*, and *Enterococcus faecalis*) to ferment DG and partially replaced the concentrate when feeding the finishing cattle. We explored the effects of feeding PFDG on average daily gain (ADG), serum antioxidant capacity, immune status, and rumen enzyme activity and further revealed the rumen microbiome and metabolome profiling of finishing cattle using 16S rDNA amplicon high-throughput sequencing and liquid chromatography–mass spectrometry (LC-MS) technology, respectively. This experiment is expected to contribute to revealing the effects of PFDG on rumen microbial status and serum physiological indices in finishing cattle.

## 2. Materials and Methods

### 2.1. Preparation of PFDG

The Moutai-flavored DG used in this study was obtained from the Kweichow Moutai Group in Moutai Town, Renhuai City, Guizhou Province, China. The main ingredients of DG are distilled sorghum and wheat, which are byproducts of brewing processes. *Lactobacillus plantarum* (ACCC11095) and *Aspergillus Niger* (CICC2377) used in the present study were provided by the Shanghai Bioresource Collection Center (Shanghai, China). *Saccharomyces cerevisiae* and *Enterococcus faecalis* were provided by the Institute of Animal Disease, Guizhou University (Guizhou, China). First, the Moutai-flavored DG was mixed with 3.5% (*m*/*m*) of baking soda, followed by 6% (*m*/*m*) corn meal, 5% (*m*/*m*) wheat bran, and 12% (*m*/*m*) canola meal. Then, the compound probiotics liquid (the concentrations of *Enterococcus faecalis*, *Lactobacillus plantarum*, *Aspergillus niger*, and *Saccharomyces cerevisiae* were all adjusted to 1 × 10^8^ CFU/mL and mixed at a ratio of 1:1:1:1 (*v*:*v*:*v*:*v*)) were inoculated into the DG at 8% (*v*/*m*). Finally, the DG was packed into 900 L plastic bucket silos, compacted with a shovel, and fermented for 5 days to produce PFDG. The plastic bucket silos were stored at room temperature (28–33 °C), away from light. Before fermentation, the crude protein (CP) content of DG was 21.34%, the neutral detergent fiber (NDF) content was 59.88%, and the acid detergent fiber (ADF) content was 32.45%. After 5 days of fermentation, the CP content of PFDG was 24.74%, the NDF content was 42.03%, and the ADF content was 22.79% (CP content was determined by the Kjeldahl method; NDF and ADF content were determined by the filter bag method). Meanwhile, during fermentation, the number of viable bacteria in PFDG was as follows: the *lactic acid bacteria (Lactobacillus plantarum* and *Enterococcus faecalis)* content on day 1, day 3, and day 5 was 9.00 × 10^6^ CFU/g, 8.333 × 10^7^ CFU/g, and 4.7 × 10^9^ CFU/g, respectively; the *Saccharomyces cerevisiae* contents on day 1, day 3, and day 5 were 2.907 × 10^5^ CFU/g, 8.567 × 10^5^ CFU/g, and 1.63 × 10^6^ CFU/g, respectively; the *Aspergillus niger* content on day 1, day 3, and day 5 was 3.733 × 10^5^ CFU/g, 4.533 × 10^3^ CFU/g, and 0, respectively (the viable count of mold and yeast was determined according to GB 4789.15-2016 [20], and the viable count of *lactic acid bacteria* was determined according to GB 4789.35-2016) [21].

### 2.2. Animals, Diets, and Experimental Design

This study was conducted at the finishing cattle breeding base in Aotian Village, Pingyuan Town, Dejiang County, Guizhou Province. A total of 30 healthy (without infection of *Brucella*, *Mycobacterium tuberculosis*, foot-and-mouth disease virus, or lumpy skin disease virus) Simmental crossbred steers (265.0 ± 22.48 kg), with similar body conditions and ages (6.5-month-old), were used in this study. A total of 30 Simmental crossbred cattle were randomly divided into 3 groups, with 10 head per group, and the diet treatments consisted of the Control group, without PFDG supplementation; the PFDG-15% group, with 15% PFDG substituting for 15% concentrate; and the PFDG-30% group, with 30% PFDG substituting for 30% concentrate. These cattle were housed in three different pens, and each cow was provided with a feeder. The ingredients and nutrient contents of the diets are shown in Table 1. The diets were mixed thoroughly to yield a total mixed ration (TMR), and the cattle were fed twice daily at 9:00 a.m. and 4:30 p.m., respectively. The study began on 1 June 2022, with 15 d as the adopting period and 45 d as the experiential period. Water was freely available during the entire period.

### 2.3. Sample Collection and Pre-Treatment

All experimental cattle were weighed before the morning feeding on days 0 and 45 of the experimental period to determine the initial body weight (IBW) (kg) and final body weight (FBW) (kg), and the ADG (kg/d) was calculated using the following formula: ADG (kg/d) = (FBW − IBW)/experimental period (45 days). At the end of the experimental period (day 45), before the morning feeding, six experimental cattle were randomly selected from each group, and blood and rumen fluid samples were collected. Blood samples were harvested from the jugular vein into 5 mL vacutainer tubes; after centrifugation at 4 °C and 1000× *g* for 10 min, the serum was obtained. All serum samples were frozen at −80 °C until analysis. Rumen fluid was drawn with a syringe by inserting the rumen sampler through the mouth into the rumen, both of which were thoroughly cleaned using fresh warm water between sample collections. The first 10 to 15 mL of the sample from each animal was discarded to avoid contamination from saliva. A subsample of 10 mL of rumen fluid was filtered through four layers of sterile gauze. Then, 5 mL of rumen fluid was centrifuged at 1000× *g* at 4 °C for 10 min; subsequently, the supernatant was immediately frozen in liquid nitrogen and then stored at −80 °C until rumen enzyme activity analysis. The rest of the rumen fluid was immediately frozen in liquid nitrogen and then stored at −80 °C until analysis.

### 2.4. Serum Parameter Analysis

Four samples from each group were randomly selected from the pre-collected serum samples and analyzed according to the serum parameters immediately after thawing. The serum levels of malondialdehyde (MDA), immunoglobulin G (IgG), immunoglobulin A (IgA), interferon-γ (IFN-γ), interleukin-2 (IL-2), interleukin 4 (IL-4), interleukin 10 (IL-10), interleukin-1β (IL-1β), interleukin 6 (IL-6), and tumor necrosis factor-α (TNF-α), as well as the activities of serum superoxide dismutase (SOD), glutathione peroxidase (GSH-Px) and catalase (CAT), were quantified using ELISA kits (Shanghai Kexing Trading Co., Ltd., Shanghai, China). Meanwhile, serum total antioxidant capacity (T-AOC) levels were determined via the micromethod and using a commercial assay kit (Shanghai Kexing Trading Co., Ltd., Shanghai, China). The protocols were carried out in strict accordance with the manufacturer’s instructions.

### 2.5. Microbial Analysis of the Rumen Fluid

Four samples were randomly selected from each group of pre-collected rumen fluid samples for microbial analysis. The total genomic DNA of the rumen fluid samples was extracted using the MagPure Soil DNA LQ Kit (Magen D6356-02, Shanghai, China). The integrity of DNA was assessed via agarose gel electrophoresis. Genomic DNA was used as a template for PCR amplification. The V3-V4 gene region of the bacteria 16S rDNA gene was PCR amplified with primers 343F (5′-TACGGRAGGCAGCAG-3′) and 798R (5′-AGGGTATCTAATCCT-3′). The PCR products were purified using magnetic beads after electrophoresis detection, and Qubit quantification was performed on the PCR products after purification. Sequencing was performed on Illumina’s Miseq PE300 platform according to the standard protocols by Oebiotech Technology Co., Ltd. (Shanghai, China). Using the QIIME bioinformatics platform, quality screening and filtering were carried out to obtain high-quality sequences. Data were analyzed and visualized for graphing using R (version 3.5.0). α diversity indicators between the Control group vs. the PFDG-15% group and the Control group vs. the PFDG-30% group were analyzed for differences using Wilcoxon’s signed-rank test, which were identified based on sequences with the lowest 100% set (amplicon sequence variant, ASV). Principal coordinate analysis (PCoA) and non-metric multidimensional scaling (NMDS) were utilized to investigate the beta-diversity analysis of colony distribution similarity, and the one-way ANOVA was used to analyze the difference in species between the Control, PFDG-15%, and PFDG-30% groups. The values were considered significantly different at *p* < 0.05.

### 2.6. LC-MS Analysis of the Rumen Fluid

Six samples per group were selected for LC-MS analysis of rumen metabolites. Briefly, rumen fluid metabolites were first extracted using the extraction liquid (methanol:acetonitrile = 2:1 (*v*:*v*)). After extraction, the samples were treated with mixed liquid (methanol:water = 1:4 (*v*:*v*)); finally, the supernatant was transferred to LC injection vials and stored at −80 °C until LC-MS analysis was performed. LC-MS was performed using a liquid mass spectrometry system consisting of an ACQUITY UPLC I-Class plus (Waters Corporation, Milford, MA, USA) ultra-high performance liquid tandem QE (Thermo Fisher Scientific, Waltham, MA, USA) high-resolution mass spectrometer, and the specific experimental procedures were conducted in strict accordance with the experimental instructions of Oebiotech Technology Co., Ltd. (Shanghai, China). Progenesis QI v2.3 software (Nonlinear Dynamics, Newcastle, UK) was used to process the originally collected data, including baseline filtering and peak identification, and it was also used to classify metabolites. The processed data were normalized relative to the total peak intensity and imported into SIMCA-P (Version 14.1, Umetrics, Umea, Sweden) for multivariate data analysis, including principal component analysis (PCA), partial least squares discriminant analysis (PLS-DA), and orthogonal partial least squares discriminant analysis (OPLS-DA), to distinguish the overall differences in metabolics among the groups. The VIP (variable importance in the projection) value of each variable in the PLS-DA model was calculated according to VIP > 1 and *p* < 0.05 to screen significantly different metabolites. Metabo Analyst 5.0, based on the KEGG database, was used for statistical analysis and the determination of metabolite metabolism pathways in the rumen fluid of different groups. Finally, the data for the metabolites in the rumen fluid of four cattle in each group were randomly selected for correlation analysis with the rumen microbial data of four other cattle.

### 2.7. Rumen Enzyme Activity Analysis

Four samples from each group were randomly selected from the pre-collected rumen supernatant samples. They were thawed and immediately analyzed for the activities of cellulase, amylase, lipase, and protease using ELISA kits (Shanghai Kexing Trading Co., Ltd., Shanghai, China). The protocols were carried out in strict accordance with the manufacturer’s instructions.

### 2.8. Statistical Analysis

Data for daily weight gain, rumen enzyme activity, and serum indexes were analyzed using SPSS 20.0 software (SAS Inc., Chicago, IL, USA). One-way ANOVA and Duncan’s test were used to determine the differences between groups. The effect was considered significant when *p* < 0.05. Data are expressed as mean ± SEM. GraphPad Prism 8 (San Diego, CA, USA) was used to generate the bar plots. Spearman’s correlation analysis of differential microbiota using daily weight gain, enzyme activity, antioxidant index, immune index, and metabolites was computed using R (Version 3.2.4), and the values were considered significantly different at *p* < 0.05.

## 3. Results

### 3.1. Effect of Feeding Varying Levels of Probiotic-Fermented Distillers Grain on the ADG of Finishing Cattle

The results showed that compared with the Control group, the ADG of cattle in the PFDG-15% group showed no significant difference, while the ADG of cattle in the PFDG-30% group was significantly decreased (*p* = 0.005) (Table 2).

### 3.2. Effect of Feeding with Varying Levels of Probiotic-Fermented Distillers Grain on the Serum Antioxidant Indexes of Finishing Cattle

**Figure 1:**
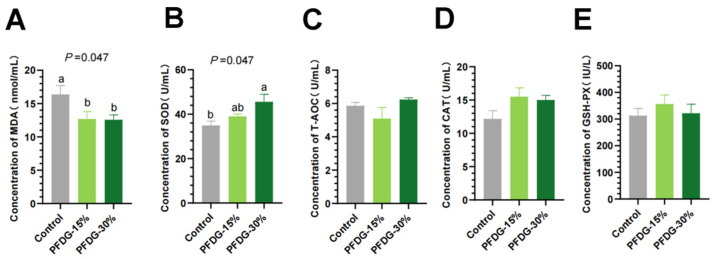
Effect of feeding with varying levels of probiotic-fermented distillers grain on the serum antioxidant indexes of finishing cattle. (**A**–**E**) Concentration of serum antioxidant indices, MDA = malondialdehyde; SOD = superoxide dismutase; T-AOC = total antioxidant capacity; CAT = catalase; GSH-Px = glutathione peroxidase. Control, PFDG-15%, and PFDG-30% represent the group without PFDG supplementation, the group with 15% PFDG substituting for 15% concentrate, and the group with 30% PFDG substituting for 30% concentrate, respectively. Samples for each experimental group consisted of serum samples from four cattle (*n* = 4). ^a,b^ Within a row, different lowercase letters represent significant differences (*p* < 0.05).

The serum antioxidant indexes of finishing cattle are presented in Figure 1. Compared with the Control group, the serum MDA content in the PFDG-15% and PFDG-30% groups was significantly decreased (*p* = 0.047) (Figure 1A). SOD activity in the serum of the PFDG-30% group was significantly increased (*p* = 0.047) (Figure 1B), but the differences in the serum levels of T-AOC, CAT and GSH-Px in the three groups were not statistically significant (*p* > 0.05) (Figure 1C–E).

### 3.3. Effect of Feeding with Varying Levels of Probiotic-Fermented Distillers Grain on the Immune Status of Finishing Cattle

**Figure 2:**
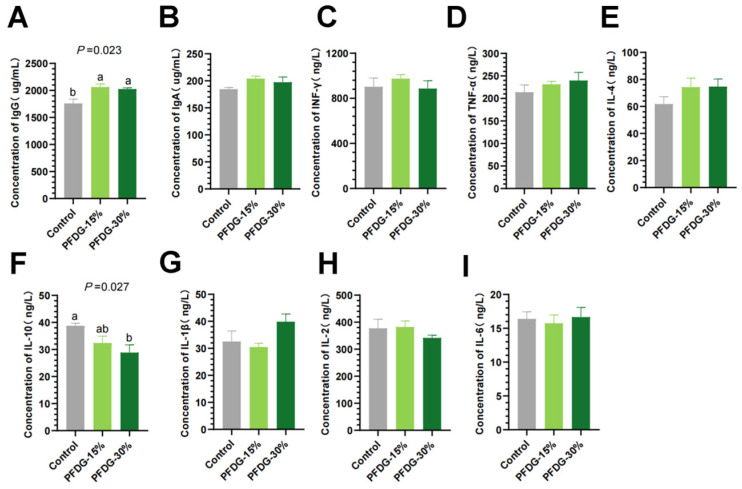
Effect of feeding with varying levels of probiotic-fermented distillers grain on the immune status of finishing cattle. Control, PFDG-15%, and PFDG-30% represent the group without PFDG supplementation, the group with 15% PFDG substituting for 15% concentrate, and the group with 30% PFDG substituting for 30% concentrate, respectively. Samples for each experimental group consisted of serum samples from four cattle (*n* = 4). ^a,b^ Within a row, different lowercase letters represent significant differences (*p* < 0.05). (**A**–**I**) Concentration of serum immune indices, IgG = immunoglobulin G; IgA = immunoglobulin A; INF-γ = interferon-γ; IL-4 = interleukin 4; TNF-α = tumor necrosis factor-α; IL-10 = interleukin 10; IL-1β = interleukin-1β; IL-2 = interleukin-2; IL-6 = interleukin 6.

The results for the cattle serum immune indexes were presented in Figure 2. We found that, compared with the Control group, the serum IgG contents in the PFDG-15% and PFDG-30% groups were significantly increased (*p* = 0.023) (Figure 2A), while the serum IL-10 contents in the PFDG-30% group were significantly decreased (*p* = 0.027) (Figure 2F). In addition, the differences in the serum levels of IgA, IFN-γ, IL-4, TNF-α, IL-1β, IL-2, and IL-6 in the three groups were not statistically significant (*p* > 0.05) (Figure 2B–E,G–I).

### 3.4. Effect of Feeding with Varying Levels of Probiotic-Fermented Distillers Grain on the Composition of Rumen Microbiota in Finishing Cattle

**Figure 3:**
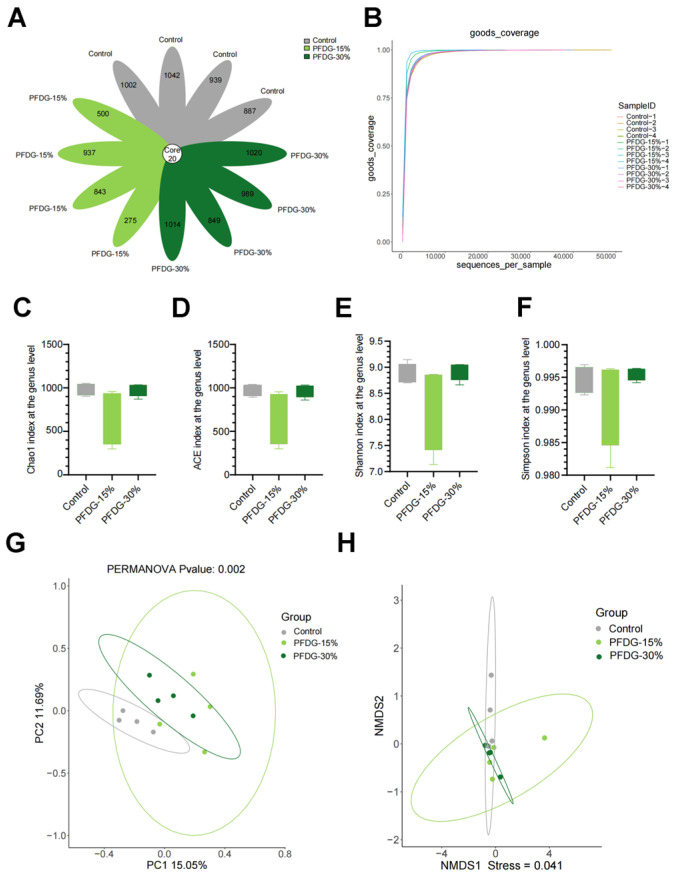
Effect of feeding with varying levels of probiotic-fermented distillers grain on rumen microbiota alpha and beta diversity in finishing cattle. (**A**) The Venn analysis of amplicon sequence variants (ASV). (**B**) Rarefaction curve of rumen microbiota. (**C**–**F**) Alpha diversity at the genus level. Whiskers represent the maximum and minimum. (**G**) Principal coordinate analysis (PCoA) based on ASV horizontal Bray–Curtis distance (*p* = 0.002). (**H**) Horizontal non-metric multidimensional scale (NMDS) (stress = 0.041); Control, PFDG-15%, and PFDG-30% represent the group without PFDG supplementation, the group with 15% PFDG substituting for 15% concentrate, and the group with 30% PFDG substituting for 30% concentrate, respectively. Samples for each experimental group consisted of rumen fluid samples from four cattle (*n* = 4).

We further analyzed the changes in the rumen microbiota in the Control, PFDG-15%, and PFDG-30% groups. A total of 965,866 valid sequences were detected, and 10,537 ASVs (amplicon sequence variants) were obtained by clustering according to the criteria of 100% sequence similarity. Venn analysis revealed that 20 ASVs were shared between the three groups (Figure 3A). Rarefaction curve results also suggested that these curves flatten out as the sequencing depth deepens, indicating that there were sufficient sequencing data for detecting all species (Figure 3B). The results of α-diversity analysis showed no statistically significant effect (*p* > 0.05) on the Chao1, ACE, Shannon, and Simpson elements of rumen microbiota in the PFDG-15% and PFDG-30% groups compared to that in the Control group (Figure 3C–F). This shows that feeding with PFDG had little effect on rumen microbiota diversity in finishing cattle. To compare the distribution of rumen microbiota in different groups, we performed beta diversity analysis using PCoA and NMDS. The results showed that the rumen microbiota of the Control, PFDG-15%, and PFDG-30% groups was obviously separated (Figure 3G, *p* = 0.002), and the diversity of the NMDS groups was also different (Figure 3H, Stress = 0.041), indicating that the grouping effect was better.

**Figure 4:**
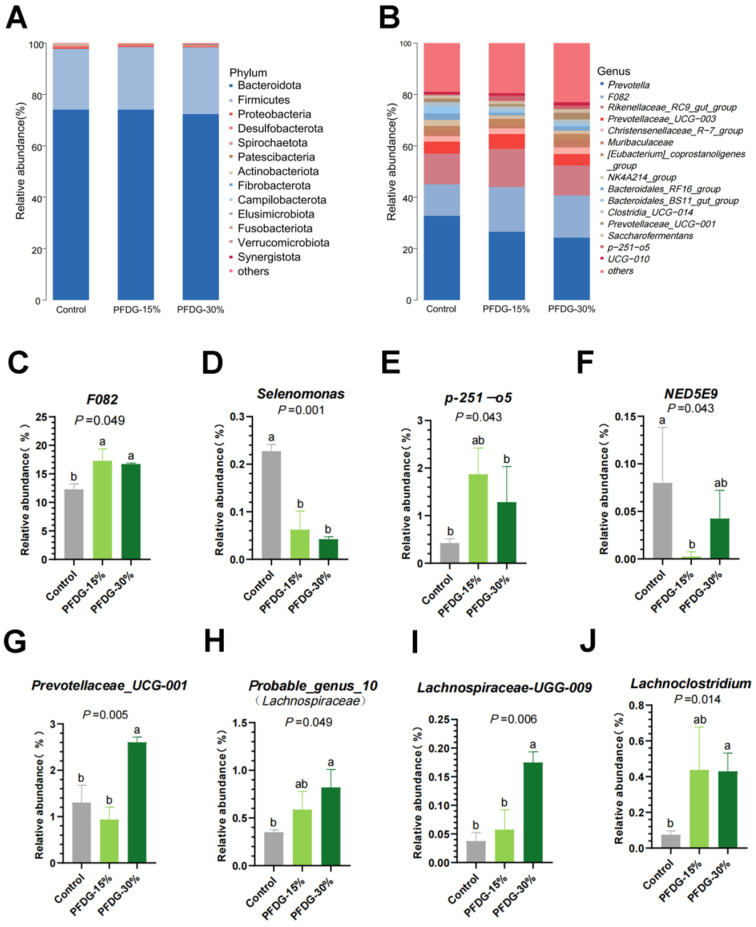
Effect of feeding with varying levels of probiotic-fermented distillers grain on the relative abundance of rumen microbiota in finishing cattle. (**A**) Relative abundance of rumen bacteria at the phylum level. (**B**) Relative abundance of rumen bacteria at the genus level. (**C**–**J**) Relative abundance of differential bacteria among groups at the genus levels. Control, PFDG-15%, and PFDG-30% represent the group without PFDG supplementation, the group with 15% PFDG substituting for 15% concentrate, and the group with 30% PFDG substituting for 30% concentrate, respectively. Samples for each experimental group consisted of rumen fluid samples from four cattle (*n* = 4). ^a,b^ Within a row, different lowercase letters represent significant differences (*p* < 0.05).

The analysis of rumen microbiota composition showed that, at the phylum level, *Bacteroidetes* and *Firmicutes* were the dominant bacteria in the rumen in the Control, PFDG-15%, and PFDG-30% groups, followed by *Proteobacteria*, and there was no statistical significance in their relative abundance (*p* > 0.05) (Figure 4A). At the genus level, the *Prevotella*, *F082*, and *Rikenellaceae_RC9_gut_group* were the dominant bacteria in all groups (Figure 4B). Further analysis found that there was no significant difference in rumen bacteria at the phylum level among the three groups; however, at the genus level, compared with the Control group, the relative abundance of the dominant bacterium *F082* in the PFDG-15% and PFDG-30% groups was significantly increased (*p* = 0.049), and the relative abundance of S*elenomonas* in the PFDG-15% and PFDG-30% groups was significantly decreased (*p* = 0.001) (Figure 4D). In addition, we also found that compared to the Control group, the relative abundance of *p_251_o5* significantly increased in the PFDG-15% group (*p* = 0.043) (Figure 4E), while the relative abundance of *NED5E9* significantly decreased in the PFDG-15% group (*p* = 0.044) (Figure 4F). The relative abundance of *Prevotellaceae_UCG-001* (*p* = 0.005), *probable_genus_10 (Lachnospiraceae*) (*p* = 0.049), *Lachnospiraceae_UGG-009* (*p* = 0.006), and *Lachnoclostridium* (*p* = 0.014) significantly increased in the PFDG-30% group (Figure 4G–J). In addition, we also found that the relative abundance of *Prevotellaceae_UCG-001* and *Lachnospiraceae_UGG-009* was significantly higher in the PFDG30% group than in the PFDG15% group.

### 3.5. Effect of Feeding with Varying Levels of Probiotic-Fermented Distillers Grain on hte Rumen Metabolome in Finishing Cattle

**Figure 5:**
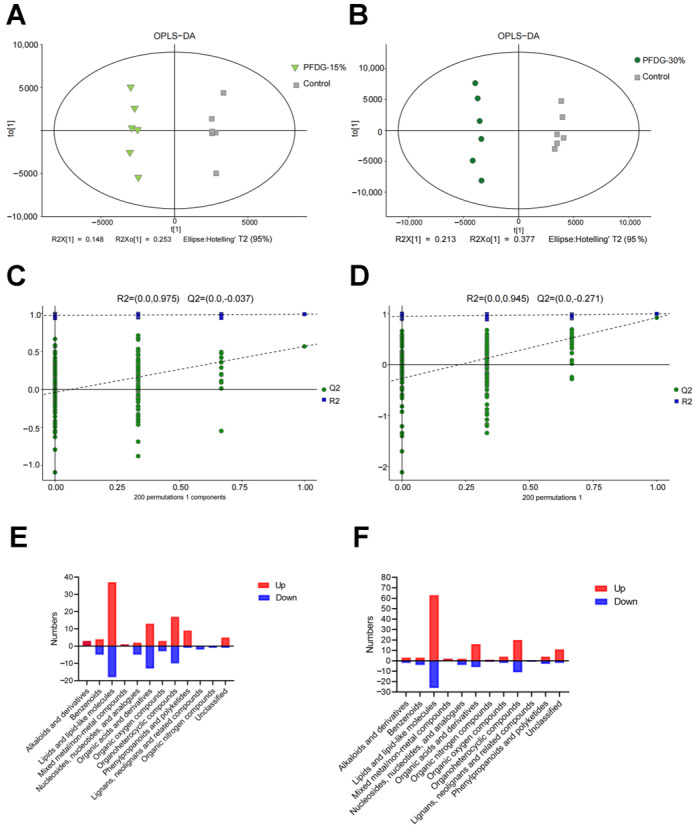
The effect of feeding with varying levels of probiotic-fermented distillers grain on the rumen metabolites in finishing cattle. (**A**,**B**) Score plot of orthogonal partial least squares—discriminant analysis (OPLS-DA) for PFDG-15% vs. Control and PFDG-30% vs. Control, respectively. (**C**,**D**) Results of response permutation testing for PFDG-15% vs. Control and PFDG-30% vs. Control, respectively. R2 and Q2 are the cumulative explanatory ability and predictive ability of the model, respectively. (**E**,**F**) Classification of rumen differential metabolites for PFDG-15% vs. Control and PFDG-30% vs. Control, respectively. (VIP > 2.0, *p* < 0.05). Control, PFDG-15%, and PFDG-30% represent the group without PFDG supplementation, the group with 15% PFDG substituting for 15% concentrate, and the group with 30% PFDG substituting 30% for concentrate, respectively. Samples for each experimental group consisted of rumen fluid samples from six cattle (*n* = 6).

The PCA score plot showed that the rumen fluid samples of the three groups were all located in the 95% Hotelling’s T2 elliptical region; these data are worthy of further study (Figure S1). Therefore, we conducted OPLS-DA and response permutation testing. The OPLS-DA score plots, which show a clear difference between the Control, PFDG-15%, and PFDG-30% groups, showed significant separation between the groups (Figure 5A,B). The results of response permutation testing (Control group vs. PFDG-15% group, R2Y = 0.975, Q2Y = −0.037 < 0; Control group vs. PFDG-30% group, R2Y = 0.945, Q2Y = −0.271 < 0) indicate that the OPLS-DA model is stable, reliable, and valid (Figure 5C,D).

After analysis by LC-MS, differential metabolites were screened according to VIP > 1 and *p* < 0.05. The results showed that there were 488 differential metabolites in the PFDG-15% group and 618 differential metabolites in the PFDG-30% group compared with the Control group (Tables S1 and S2). For more convenient analysis, we raised the screening criteria and screened the differential metabolites, according to VIP > 2, *p* < 0.05. We found 153 differential metabolites in the PFDG-15% group and 191 differential metabolites in the PFDG-30% group compared to the Control group. These rumen differential metabolites were classified according to the properties of the compounds. It was determined that the differential metabolites of the PFDG-15% and PFDG-30% groups mainly included lipids and lipid-like molecules, organoheterocyclic compounds, and organic acids and derivatives (Figure 5E,F).

**Figure 6:**
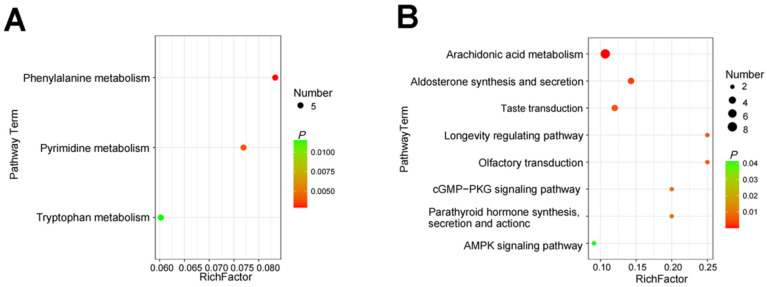
Effect of feeding with varying levels of probiotic-fermented distillers grain on the rumen metabolic pathway in finishing cattle. (**A**,**B**) Pathway analysis of significant differences in rumen metabolites of PFDG-15% vs. Control and PFDG-30% vs. Control, respectively. Control, PFDG-15%, and PFDG-30% represent the group without PFDG supplementation, the group with 15% PFDG substituting for 15% concentrate, and the group with 30% PFDG substituting for 30% concentrate, respectively. The samples for each experimental group consisted of rumen fluid samples from six cattle (*n* = 6).

Based on the above results, we further annotated the metabolic pathways involved in the differential metabolites using the KEGG database. The results showed that compared with the Control group, the PFDG-15% group significantly enriched three metabolic pathways, including phenylalanine metabolism, pyrimidine metabolism, and tryptophan metabolism (Figure 6A). The PFDG-30% group significantly enriched eight metabolic pathways, including arachidonic acid metabolism; aldosterone synthesis and secretion; taste transduction; the longevity-regulating pathway; olfactory transduction; the cGMP-PKG signaling pathway; parathyroid hormone synthesis, secretion, and action; and the AMPK signaling pathway (Figure 6B), in which metabolites were mainly enriched in regards to arachidonic acid metabolism.

### 3.6. Effect of Feeding with Varying Levels of Probiotic-Fermented Distillers Grain on Rumen Enzyme Activity of Finishing Cattle

**Figure 7:**
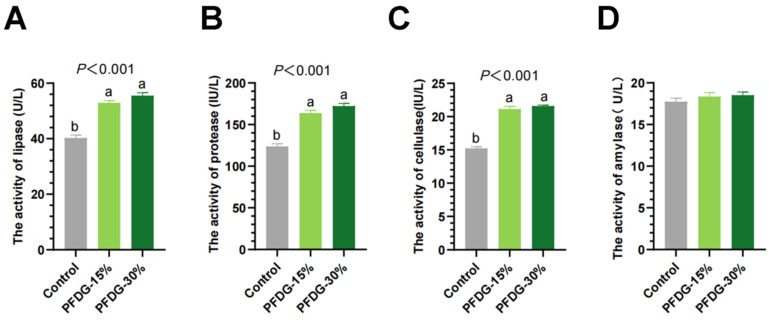
Effect of feeding with varying levels of probiotic-fermented distillers grain on rumen enzyme activity of finishing cattle. (**A**–**D**) The activities of lipase, protease, cellulase and amylase in rumen. Control, PFDG-15%, and PFDG-30% represent the group without PFDG supplementation, the group with 15% PFDG substituting for 15% concentrate, and the group with 30% PFDG substituting for 30% concentrate, respectively. The samples for each experimental group consisted of rumen fluid samples from four cattle (*n* = 4). ^a,b^ Within a row, different lowercase letters represent significant differences (*p* < 0.05).

The results of rumen enzyme activity showed that both the PFDG-15% and PFDG-30% groups significantly increased rumen lipase, protease, and cellulase activities (*p* < 0.01) compared to the levels in the Control group (Figure 7A–C), while the effect of feeding with PFDG on amylase activity was not significant (*p* = 0.426) (Figure 7D).

### 3.7. Correlation Analysis of Rumen Microbiota according to ADG, Enzyme Activity, Antioxidant Indexes, Immune Indexes, and Metabolites

**Figure 8:**
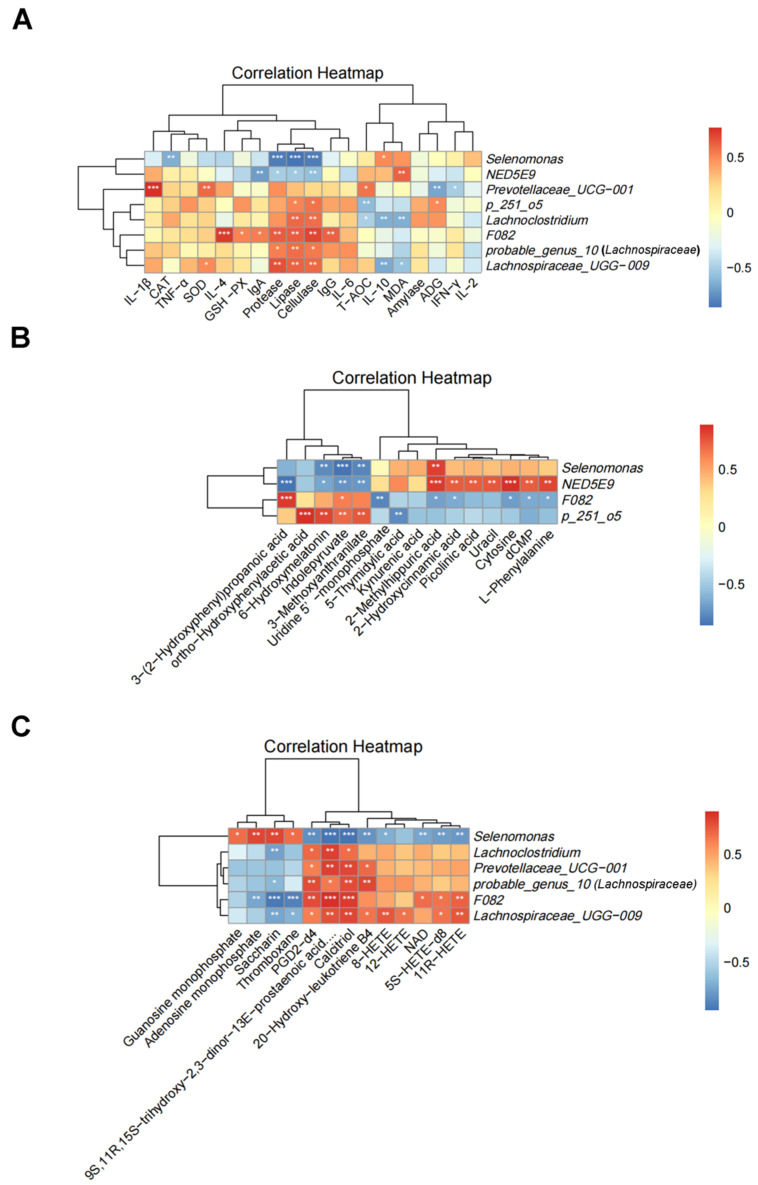
Correlation analysis of rumen microbiota with ADG, enzyme activity, antioxidant indexes and immune indexes, and metabolites. Red and blue colors represent positive and negative correlations, respectively, and color gradation indicates the size of the correlation coefficient. Significant correlations are noted by: * 0.01 < *p* ≤ 0.05; ** 0.01 < *p* ≤ 0.001; and *** *p* < 0.001 (*n* = 4). (**A**) Correlation analysis of rumen microbiota with ADG, enzyme activity, and antioxidant indexes and immune indexes. ADG = average daily gain; IgG = immunoglobulin G; IgA = immunoglobulin A; INF-γ = interferon-γ; IL-4 = interleukin 4; TNF-α = tumor necrosis factor-α; IL-10 = interleukin 10; IL-1β = interleukin-1β; IL-2 = interleukin-2; and IL-6 = interleukin 6. (**B**,**C**) Heat map of correlation between differential bacteria and metabolites in key metabolic pathways in the Control group vs. the PFDG-15% group and the Control group vs. the PFDG-30% group, respectively. Control, PFDG-15%, and PFDG-30% represent the group without PFDG supplementation, the group with 15% PFDG substituting for 15% concentrate, and the group with 30% PFDG substituting for 30% concentrate, respectively.

The correlation results showed that the differential bacteria in the rumen was tightly related to ADG, serum antioxidant capacity, immunity, and rumen enzyme activity (Figure 8A). For example, the level of ADG was positively correlated with the relative abundance of *p-251-o5 (Lachnospiraceae*) (*p* = 0.029); the serum antioxidant index MDA content was negatively correlated with the relative abundance of *Lachnospiraceae_UGG-009* (*p* = 0.023) and positively correlated with *NED5E9* (*p* < 0.01); the serum immune indexes IgA and IgG contents were positively correlated with the relative abundance of *F082* (*p* < 0.01); the content of anti-inflammatory factor IL-4 was positively correlated with the relative abundance of *F082* (*p* < 0.001); and the content of pro-inflammatory factor IL-1β was positively correlated with the relative abundance of *Prevotellaceae_UCG-001* (*p* < 0.001). The relative abundance of *F082*, *probable_genus_10 (Lachnospiraceae*), and *Lachnospiraceae_UGG-009* was positively correlated with protease, lipase, and cellulase (*p* < 0.05). However, the relative abundance of *Selenomonas* and *NED5E9* was negatively correlated with the activities of protease, lipase, and cellulase (*p* < 0.05).

As shown in Figure 8B, the differential bacteria in the rumen were significantly correlated with metabolites in the key metabolic pathways in the PFDG-15% group, i.e., the relative abundance of *F082* was positively correlated with the levels of 3-(2-Hydroxyphenyl) propanoic acid and Indolepyruvate (*p* < 0.05), but negatively correlated with the contents of Cytosine, dCMP, and Uridine 5′-monophosphate (*p* < 0.05). In addition, as shown in Figure 8C, the differential bacteria in the rumen were significantly correlated with metabolites in key metabolic pathways in the PFDG-30% group. For example, the relative abundances of *Lachnoclostridium*, *Lachnospiraceae_UGG-009*, and *F082* were positively correlated with the contents of PGD2-d4 and Calcitriol *(p* < 0.05), but was negatively correlated with saccharin content *(p* < 0.05).

## 4. Discussion

The DG is rich in nutrients and is often added to animal feed. ADG is an important indicator of animal growth and development [22]. However, the effects of DG on the ADG of livestock are complicated. Reis et al. [23] found that feeding with 21% low-fat dry DG increased the ADG of bulls, but Larson et al. [24] noted that feeding with 40% DG made no significant difference in the ADG of cattle. Sarturi et al. [25] also found that feeding with 40% dry DG even slightly reduced ADG in cattle. In our study, there was no significant difference in ADG in the PFDG-15% group and a significant decrease in ADG in the PFDG-30% group compared to that of the Control group. This may be related to the animals’ own tolerance levels to PFDG. The PFDG contains ethanol and produces substances such as propionic acid during fermentation; elevated levels of both ethanol and propionic acid cause a pungent odor and decrease feed palatability and animal appetite, thus reducing ADG [26]. We acknowledge the limitations of this study, including the lack of detailed intake data to estimate nutrient utilization efficiency or feed conversion ratio, which could have provided a more comprehensive understanding of the relationship between PFDG and ADG. We will focus on avoiding such problems in future research.

Probiotics including *Lactobacillus* and *Bifidobacterium* may scavenge reactive oxygen species via their antioxidant system [27], enzyme-producing activity, and the regulation of host gastrointestinal microbiota to regulate the redox status of the host [12]. In addition, studies have proven that DG is rich in polyphenolic compounds with strong bioactive properties, including total phenols and anthocyanins [28], and exhibits strong antioxidant activities [29]. Li et al. [30] found that dried distillers’ grains containing solubles (DDGS) increased the activities of CAT, T-SOD, and GSH-Px in the serum of dairy cows. In our study, feeding PFDG increased the activities of GSH-Px, SOD, and CAT in serum, to some extent, and decreased the content of MDA in serum, among which the activity of SOD and the content of MDA were significantly different, which was basically consistent with the results of Li et al. [30], indicating that feeding with PFDG increased the antioxidant capacity of finishing cattle.

Antioxidant capacity reflects the health status of the host, to a certain extent [31]. Therefore, we further measured serum immunoglobulin and inflammatory factor levels in finishing cattle. IgG and IgA can activate, complement, and regulate the immune response, playing an important role in improving the animal’s immune status [32]. Studies have shown that when the body’s IgG or IgA is increased, it can boost the body’s immunity [33,34]. The present study has shown that adding PFDG to the basal diet can increase the level of IgA and promote improved immunity in finishing cattle [35]. In our study, it was found that compared with the Control group, the serum IgA and IgG levels of finishing cattle fed with PFDG were higher, indicating that PFDG improved their immunity, a result which was consistent with that of Cheng et al. [35]. IL-10 itself can repress proinflammatory responses and limit unnecessary tissue disruptions caused by inflammation [36]. IL-1β is a proinflammatory factor of innate immunity, and it can cause localized inflammation by binding to the IL-1 receptor 1 [37]. We found that compared with the Control group, the content of IL-10 was significantly reduced, and the content of IL-1β was increased in the PFDG-30% group, implying that inflammation might occur in finishing cattle. Barekatain et al. [38] have suggested that the dietary supplementation of a high level of sorghum DDGS can increase susceptibility to the necrotizing enterocolitis of broilers. Therefore, it is inferred that in this study, the decrease in IL-10 and the increase in IL-1β in the PFDG-30% group may be related to the added amount of PFDG; thus, exploring the appropriate feeding level for the application of DG as animal feeds is important.

Rumen, as a unique digestive organ of ruminants, is inhabited by a large number of microorganisms which are closely related to the digestive and health status of animals [39]. Through the analysis of rumen microorganisms, we found that feeding with PFDG had little effect on rumen microbial diversity, which was consistent with the results of Song et al. [40]. At the same time, feeding with PFDG had little effect on the change in bacterial abundance at the phylum level, which was consistent with the results of previous studies [41]. This phenomenon may be related to the self-regulatory adaptive capacity of rumen microbiota. However, at the genus level, compared with the Control group, the numbers of dominant bacteria *F082* in the PFDG-15% and PFDG-30% groups were significantly increased, while those of *Selenomonas* were significantly decreased. It is worth noting that *F082* and *Selenomonas* are associated with propionic acid production [42]. *Selenomonas* can decarboxylate succinate to propionic acid [43]. *Selenomonas* has been shown to promote propionic acid production in the rumen of cattle [44]. The regulatory role of *F082* is still unclear, but studies have shown that higher molar proportions of propionate were related to higher relative abundances of unidentified *F082* during rumen fermentation in cattle [45,46]. Propionic acid is a type of short-chain fatty acid (SCFA), and SCFAs were demonstrated to produce anti-oxidant and immunity-improving effects. A study has shown that SCFAs can improve the antioxidant capacity of animals by reducing MDA levels [47]. Alternatively, SCFAs have been shown to mediate pro-inflammatory effects by upregulating B-cell metabolism, thereby increasing the systemic production of IgG and IgA [48]. This may be related to the decrease in MDA and the increase in IgG and IgA in the PFDG-15% and PFDG-30% groups in this study. Some studies have also indicated that *Selenomonas* may contribute to the development of periodontal disease, bacteremia, and asthma [49,50]. This suggested that the PFDG diet regulates the rumen microbiota by increasing the abundance of bacteria associated with propionic acid production (*F082*) and decreasing the abundance of bacteria associated with disease (*Selenomonas*).

*Lachnospiraceae_UGG-009* and *Prevotellaceae_UCG-001* are cellulose-decomposing bacteria, and they digest high-fiber diets into nutrients that are easily absorbed by cattle [51,52]. Our results showed that the relative abundance of cellulose-degrading bacteria in the rumen of finishing cattle was increased after feeding with PFDG. In addition, we also found a significant increase in the relative abundance of *Prevotellaceae_UCG-001* and *Lachnoclostridium* in the PFDG-30% group, which have been shown to be associated with inflammation [53], but the specific pro-inflammatory mechanisms have not been reported. *Prevotellaceae_UCG-001* belongs to the *Prevotellaceae* species. *Prevotellaceae* produces succinate when it digests cellulose, which promotes a pro-inflammatory response in macrophages [54]. At the same time, the relative abundance of *Prevotellaceae_UCG-001* was negatively correlated with the level of ruminal propionic acid (SCFAs with anti-inflammatory effects) in cattle [55]. *Lachnoclostridium* abundance was found to increase with inflammation induced by carcinogenesis, and decrease with inflammation suppression from probiotic administration in a mouse model [56,57]. Meanwhile, Redding et al. [58] also demonstrated that the relative abundance of *Lachnoclostridium* was significantly increased in calves with diarrheal disease caused by *Clostridioides difficile* colonization. In our study, we found that *Prevotellaceae_UCG-001* was significantly positively correlated with IL-1β, and *Lachnoclostridium* was significantly negatively correlated with IL-10. Therefore, we inferred that the decrease in IL-10 and the increase in IL-1β in the PFDG-30% group may be related to the increase in the relative abundance of *Prevotellaceae_UCG-001* and *Lachnoclostridium*, and the specific reasons require verification using subsequent experiments.

There is a dynamic association between microbiota and metabolites [59]. Our results showed that, compared with the Control group, the differential metabolites of the PFDG-15% and PFDG-30% groups were mainly lipids and lipid molecules, organoheterocyclic compounds, and organic acids and derivatives, consistent with the results of many studies on dietary changes [60,61]. KEGG analysis illustrated that phenylalanine metabolism, tryptophan metabolism, and pyrimidine metabolism were enriched in the PFDG-15% group. Phenylalanine metabolism produces higher levels of essential amino acids, such as phenylalanine and tyrosine in animals, which play important roles in processes such as protein production and glycogen synthesis [62]. Further analysis showed that ortho-Hydroxyphenylacetic acid and 3-(2-Hydroxyphenyl)propanoic acid in phenylalanine metabolism were upregulated. They were also significantly associated with differential bacteria, such as *NED5E9* or *p_251_o5*. Relevant studies have proved that ortho-Hydroxyphenylacetic acid has antibacterial effects on pathogenic bacteria [63]. 3-(2-Hydroxyphenyl)propanoic acid can inhibit liver lipid synthesis and inflammatory factors, correct intestinal microbial disorders, and maintain the intestinal barrier [64]. Both pyrimidine metabolism and tryptophan metabolism show immunomodulatory effects and have been reported to have anti-tumor and anticancer potential [65,66]. Indole and its derivatives “Indolepyruvate and 6-Hydroxymelatonin” in tryptophan metabolism were upregulated and significantly correlated with *F082* or *p_251_o5*. Indole and various indole derivatives can participate in immune regulation, enhance intestinal mucosal healing, and show anticancer potential [67]. In addition, Uridine 5′-monophosphate in pyrimidine metabolism was downregulated and significantly correlated with *F082*. Uridine 5′-monophosphate has been shown to enhance phosphatidylcholine (a biomarker for breast cancer) production in animals [68,69]. These results suggest that the PFDG-15% group may alter metabolites by regulating rumen microorganisms, which has a positive effect on phenylalanine metabolism, tryptophan metabolism, and pyrimidine metabolism.

In addition, in our study, compared with the Control group, the PFDG-30% group was significantly enriched in eight key metabolic pathways, such as arachidonic acid metabolism, aldosterone synthesis and secretion, and taste transduction, among which the metabolites were mainly enriched in arachidonic acid metabolism. Arachidonic acid metabolism is involved in various pathophysiological processes related to inflammation in the body [70,71]. Further analysis showed that 20-Hydroxy-leukotriene B4 and 12-HETE were upregulated in arachidonic acid metabolism, both of which were significantly correlated with *Lachnospiraceae_UGG-009*. Meanwhile, 20-Hydroxy-leukotriene B4 and 12-HETE have been shown to be associated with pathological processes such as inflammation, oxidative stress, and allergic reactions [72,73]. These results suggest that the PFDG-30% group may promote arachidonic acid metabolism by regulating rumen microorganisms to alter metabolites, suggesting that finishing cattle in the PFDG-30% group may be at risk for inflammation.

The microbiota produces various digestive enzymes, such as protease, lipase, and cellulase [74]. We analyzed the rumen digestive enzymes and found that the activities of protease, lipase, and cellulase in the rumen fluid of finishing cattle were significantly increased after feeding with PFDG, which was consistent with the results of previous studies showing that feeding with probiotics and probiotic fermentation products can promote the animal digestive enzyme activity and improve the efficiency of the digestion and absorption of nutrients [75,76]. Finally, via correlation analysis, we found that the relative abundance of differential bacteria in the rumen was related to changes in ADG, serum antioxidant capacity, immune status, rumen enzyme activity, and metabolites.

## 5. Conclusions

In this paper, we observed that feeding with 15% PFDG could improve the antioxidant capacity, immune status, and rumen enzyme activity, without affecting the ADG, of cattle. At the same time, it can regulate the structure of rumen microbiota by increasing the abundance of *F082* (related to propionic acid production) and fiber-degrading bacteria (*Lachnospiraceae_UGG-009* and *Prevotellaceae_UCG-001*) and reduce the abundance of the disease-related bacteria *Selenomonas*; moreover, it has a positive effect on phenylalanine metabolism, tryptophan metabolism, and pyrimidine metabolism in the rumen. However, when the supplemental level reaches 30%, the finishing cattle may develop a tolerance phenomenon, which has a negative effect on the ADG and anti-inflammatory factors and positively affects the arachidonic acid metabolism in the rumen. These results suggest that it is feasible to include PFDG at up to 15% DM, without affecting the ADG of finishing cattle.

## Figures and Tables

**Table 1:** Composition and nutrient content of the experimental diets varying in levels of PFDG ^1^ (%, on dry matter basis).

Items	Control	PFDG-15%	PFDG-30%
Ingredient			
Silo corn	55.00	55.00	55.00
PFDG	0.00	15.00	30.00
Corn	15.00	11.63	7.70
Wheat bran	11.50	6.64	2.00
Soybean meal	5.50	2.78	0.00
Rapeseed meal	9.90	6.0	2.40
Calcium carbonate, 1%	0.80	0.73	0.70
Calcium hydrogen phosphate	0.20	0.12	0.10
Sodium chloride	0.10	0.10	0.10
Sodium bicarbonate	1.00	1.00	1.00
Premix ^2^	1.00	1.00	1.00
Total	100.00	100.00	100.00
Nutrition levels ^3^			
Dry matter, DM, %	36.73	36.83	36.34
Metabolic energy, ME, MJ/kg	9.73	9.83	9.84
Crude protein, CP, %	12.82	12.53	12.45
Neutral detergent fiber, NDF, %	39.92	40.38	40.81
Acid detergent fiber, ADF, %	21.51	21.85	22.02
Calcium, Ca, /%	0.82	0.79	0.84
Phosphorus, P, %	0.43	0.48	0.45

^1^ Control, PFDG-15%, and PFDG-30% represent the group without PFDG supplementation, the group with 15% PFDG substituting for 15% concentrate, and the group with 30% PFDG substituting for 30% concentrate, respectively. ^2^ The premix provided the following amount of vitamins and minerals per kilogram: vitamin A, 100–500 KU; vitamin D3, 50–200 KIU; vitamin E, ≥500 IU; Fe (FeSO_4_), 1000–10,000 mg; Cu (CuSO_4_), 500–1500 mg; Zn (ZnSO_4_), 1000–5000 mg; Mn (MnSO_4_), 1000–7000 mg; Se (Na_2_SeO_3_), 5–20 mg; Co (CoCl_2_), 5–50 mg; I_2_(KI), 20–100 mg. ^3^ The nutrient levels are the analyzed values.

**Table 2:** Effect of feeding varying levels of probiotic-fermented distillers grain on ADG of finishing cattle. ^1^

Items	Control	PFDG-15%	PFDG-30%	*p*
IBW, kg	262.900 ± 7.009	267.100 ± 8.059	265.000 ± 6.909	0.922
FBW, kg	302.950 ± 7.707	309.800 ± 7.206	299.550 ± 7.193	0.611
ADG, kg/d	0.890 ± 0.033 ^a^	0.949 ± 0.045 ^a^	0.768 ± 0.028 ^b^	0.005

ADG = average daily gain; IBW = initial body weight; FBW = final body weight. ^a,b^ Within a row, different lowercase letters represent significant differences (*p* < 0.05). ^1^ Data are presented as mean ± SEM (*n* = 10). Control, PFDG-15%, and PFDG-30% represent the group without PFDG supplementation, the group with 15% PFDG substituting for 15% concentrate, and the group with 30% PFDG substituting for 30% concentrate, respectively.

## Data Availability

The authors confirm that the data supporting the findings of this study are available within the article and the Supplementary Materials.

## Supplementary Materials

The following supporting information can be downloaded at: https://www.mdpi.com/article/10.3390/ani13243774/s1. Figure S1: PCA score plots generated from rumen fluid metabolic profiles; Table S1: Information on differential metabolites in the Control group vs. PFDG-15% group; Table S2: Information on differential metabolites in the Control group vs. PFDG-30% group.

## References

[1] Sun Q., Gao F., Yu Z., Tao Y., Zhao S., Cai Y. (2012). Fermentation quality and chemical composition of shrub silage treated with lactic acid bacteria inoculants and cellulase additives. Anim. Sci. J..

[2] Selaledi L., Mbajiorgu C.A., Mabelebele M. (2020). The use of yellow mealworm (T. molitor) as alternative source of protein in poultry diets: A review. Trop. Anim. Health Prod..

[3] Kim S.W., Less J.F., Wang L., Yan T., Kiron V., Kaushik S.J., Lei X.G. (2019). Meeting Global Feed Protein Demand: Challenge, Opportunity, and Strategy. Annu. Rev. Anim. Biosci..

[4] Lv J., Ao X., Li Q., Cao Y., Chen Q., Xie Y. (2019). Steam co-gasification of different ratios of spirit-based distillers’ grains and anthracite coal to produce hydrogen-rich gas. Bioresour. Technol..

[5] Liu K. (2011). Chemical composition of distillers grains, a review. J. Agric. Food. Chem..

[6] Schwarz T., Przybylo M., Zapletal P., Turek A., Pabianczyk M., Bartlewski P.M. (2021). Effects of Using Corn Dried Distillers’ Grains with Solubles (cDDGS) as a Partial Replacement for Soybean Meal on the Outcomes of Pig Fattening, Pork Slaughter Value and Quality. Animals.

[7] Alimkulo Z., Velyamov M., Potoroko I., Kuanysh S., Zhumaliyeva T., Shauliyeva K. (2022). Development of Resource-Saving Biotechnology for the Production of a Feed Additive from Distiller’s Wastes with Probiotic Properties. Arch. Razi Inst..

[8] Clark P.W., Armentano L.E. (1993). Effectiveness of neutral detergent fiber in whole cottonseed and dried distillers grains compared with alfalfa haylage. J. Dairy Sci..

[9] Fan W., Sun X., Cui G., Li Q., Xu Y., Wang L., Li X., Hu B., Chi Z. (2023). A strategy of co-fermentation of distillers dried grains with solubles (DDGS) and lignocellulosic feedstocks as swine feed. Crit. Rev. Biotechnol..

[10] Wang C., Su W., Zhang Y., Hao L., Wang F., Lu Z., Zhao J., Liu X., Wang Y. (2018). Solid-state fermentation of distilled dried grain with solubles with probiotics for degrading lignocellulose and upgrading nutrient utilization. AMB Express.

[11] Mishra V., Shah C., Mokashe N., Chavan R., Yadav H., Prajapati J. (2015). Probiotics as potential antioxidants: A systematic review. J. Agric. Food Chem..

[12] Dia V.P., Wang Z., West M., Singh V., West L., de Mejia E.G. (2015). Processing method and corn cultivar affected anthocyanin concentration from dried distillers grains with solubles. J. Agric. Food Chem..

[13] Yao K.Y., Zhang T.Z., Wang H.F., Liu J.X. (2018). Upgrading of by-product from beverage industry through solid-state fermentation with Candida utilis and Bacillus subtilis. Lett. Appl. Microbiol..

[14] Kim S.K., Guevarra R.B., Kim Y.T., Kwon J., Kim H., Cho J.H., Kim H.B., Lee J.H. (2019). Role of Probiotics in Human Gut Microbiome-Associated Diseases. J. Microbiol. Biotechnol..

[15] Matthews C., Crispie F., Lewis E., Reid M., O’Toole P.W., Cotter P.D. (2019). The rumen microbiome: A crucial consideration when optimising milk and meat production and nitrogen utilisation efficiency. Gut Microbes.

[16] Gill S.R., Pop M., Deboy R.T., Eckburg P.B., Turnbaugh P.J., Samuel B.S., Gordon J.I., Relman D.A., Fraser-Liggett C.M., Nelson K.E. (2006). Metagenomic analysis of the human distal gut microbiome. Science.

[17] Paganelli A., Righi V., Tarentini E., Magnoni C. (2022). Current Knowledge in Skin Metabolomics: Updates from Literature Review. Int. J. Mol. Sci..

[18] Zengin M., Sur A., İlhan Z., Azman M.A., Tavşanlı H., Esen S., Bacaksız O.K., Demir E. (2022). Effects of fermented distillers grains with solubles, partially replaced with soybean meal, on performance, blood parameters, meat quality, intestinal flora, and immune response in broiler. Res. Vet. Sci..

[19] Wiseman M., McBride B., Li J., Wey D., Zhu J., de Lange C.F.M. (2017). Effects of steeped or fermented distillers dried grains with solubles on growth performance in weanling pigs. J. Anim. Sci..

[20] (2018). National Food Safety Standard—Food Microbiological Examination—Enumeration of Moulds and Yeasts.

[21] (2017). National Food Safety Standard—Microbiological Examination of Food—Examination of Lactic Acid Bacteria.

[22] Fang S., Chen X., Pan J., Chen Q., Zhou L., Wang C., Xiao T., Gan Q.F. (2020). Dynamic distribution of gut microbiota in meat rabbits at different growth stages and relationship with average daily gain (ADG). BMC Microbiol..

[23] Reis V.A., Reis R.A., Araujo T., Lage J.F., Teixeira P.D., Gionbelli T., Lanna D.P., Ladeira M.M. (2020). Performance, beef quality and expression of lipogenic genes in young bulls fed low-fat dried distillers grains. Meat Sci..

[24] Larson H.E., Jaderborg J.P., Paulus-Compart D.M., Crawford G.I., DiCostanzo A. (2023). Effect of substitution of distillers grains and glycerin for steam-flaked corn in finishing cattle diets on growth performance and carcass characteristics. J. Anim. Sci..

[25] Sarturi J.O., Erickson G.E., Klopfenstein T.J., Vasconcelos J.T., Griffin W.A., Rolfe K.M., Benton J.R., Bremer V.R. (2013). Effect of sulfur content in wet or dry distillers grains fed at several inclusions on cattle growth performance, ruminal parameters, and hydrogen sulfide. J. Anim. Sci..

[26] Estell R., Tellez M., Fredrickson E., Anderson D., Havstad K., Remmenga M. (2001). Extracts of Flourensia cernua reduce consumption of alfalfa pellets by sheep. J. Chem. Ecol..

[27] Singh V., Ahlawat S., Mohan H., Gill S.S., Sharma K.K. (2022). Balancing reactive oxygen species generation by rebooting gut microbiota. J. Appl. Microbiol..

[28] Lu Q., Luo Q., Li J., Wang X., Ban C., Qin J., Tian Y., Tian X., Chen X. (2022). Evaluation of the Chemical Composition, Bioactive Substance, Gas Production, and Rumen Fermentation Parameters of Four Types of Distiller’s Grains. Molecules.

[29] He R., Yang Y., Li Y., Yang M., Kong L., Yang F. (2023). Recent Progress in Distiller’s Grains: Chemical Compositions and Biological Activities. Molecules.

[30] Li Y., Zhang G.N., Fang X.P., Zhao C., Wu H.Y., Lan Y.X., Che L., Sun Y.K., Lv J.Y., Zhang Y.G. (2021). Effects of replacing soybean meal with pumpkin seed cake and dried distillers grains with solubles on milk performance and antioxidant functions in dairy cows. Animal.

[31] Guo Y., Wang Y., Liu Z., Guo X., Deng Y., Ouyang Q., Liu H., Hu S., Hu B., Li L. (2021). Effects of rearing systems on production performance, antioxidant capacity and immune status of meat ducks at different ages. Animal.

[32] Sun Y., Li X., Wang T., Li W. (2022). Core Fucosylation Regulates the Function of Pre-BCR, BCR and IgG in Humoral Immunity. Front. Immunol..

[33] Cheng Z.J., Huang H., Zheng P., Xue M., Ma J., Zhan Z., Gan H., Zeng Y., Lin R., Li S. (2022). Humoral immune response of BBIBP COVID-19 vaccination before and after the booster immunization. Allergy.

[34] Gazzinelli-Guimaraes A.C., Gazzinelli-Guimaraes P.H., Nogueira D.S., Oliveira F., Barbosa F.S., Amorim C., Cardoso M.S., Kraemer L., Caliari M.V., Akamatsu M.A. (2018). IgG Induced by Vaccination with Ascaris suum Extracts Is Protective Against Infection. Front. Immunol..

[35] Cheng Q., Xu D., Chen Y., Zhu M., Fan X., Li M., Tang X., Liao C., Li P., Chen C. (2022). Influence of Fermented-Moutai Distillers’ Grain on Growth Performance, Meat Quality, and Blood Metabolites of Finishing Cattle. Front. Vet. Sci..

[36] Ouyang W., Rutz S., Crellin N.K., Valdez P.A., Hymowitz S.G. (2011). Regulation and functions of the IL-10 family of cytokines in inflammation and disease. Annu. Rev. Immunol..

[37] Yazdi A.S., Ghoreschi K. (2016). The Interleukin-1 Family. Adv. Exp. Med. Biol..

[38] Barekatain M.R., Antipatis C., Rodgers N., Walkden-Brown S.W., Iji P.A., Choct M. (2013). Evaluation of high dietary inclusion of distillers dried grains with solubles and supplementation of protease and xylanase in the diets of broiler chickens under necrotic enteritis challenge. Poult. Sci..

[39] Zheng Y., Wang X., Yang F. (2019). Improving the Anaerobic Digestion of Switchgrass via Cofermentation of Rumen Microorganisms (Rumen Bacteria, Protozoa, and Fungi) and a Biogas Slurry. J. Agric. Food. Chem..

[40] Song C., Zhang T., Xu D., Zhu M., Mei S., Zhou B., Wang K., Chen C., Zhu E., Cheng Z. (2023). Impact of feeding dried distillers’ grains with solubles diet on microbiome and metabolome of ruminal and cecal contents in Guanling yellow cattle. Front. Microbiol..

[41] Yi S., Dai D., Wu H., Chai S., Liu S., Meng Q., Zhou Z. (2022). Dietary Concentrate-to-Forage Ratio Affects Rumen Bacterial Community Composition and Metabolome of Yaks. Front. Nutr..

[42] Han H., Zhang L., Shang Y., Wang M., Phillips C., Wang Y., Su C., Lian H., Fu T., Gao T. (2022). Replacement of Maize Silage and Soyabean Meal with Mulberry Silage in the Diet of Hu Lambs on Growth, Gastrointestinal Tissue Morphology, Rumen Fermentation Parameters and Microbial Diversity. Animals.

[43] Cibis K.G., Gneipel A., König H. (2016). Isolation of acetic, propionic and butyric acid-forming bacteria from biogas plants. J. Biotechnol..

[44] Shinkai T., Enishi O., Mitsumori M., Higuchi K., Kobayashi Y., Takenaka A., Nagashima K., Mochizuki M., Kobayashi Y. (2012). Mitigation of methane production from cattle by feeding cashew nut shell liquid. J. Dairy. Sci..

[45] Jize Z., Zhuoga D., Xiaoqing Z., Na T., Jiacuo G., Cuicheng L., Bandan P. (2022). Different feeding strategies can affect growth performance and rumen functions in Gangba sheep as revealed by integrated transcriptome and microbiome analyses. Front. Microbiol..

[46] Ma T., Wu W., Tu Y., Zhang N., Diao Q. (2020). Resveratrol affects in vitro rumen fermentation, methane production and prokaryotic community composition in a time- and diet-specific manner. Microb. Biotechnol..

[47] Sun Y., Zhou C., Chen Y., He X., Gao F., Xue D. (2022). Quantitative increase in short-chain fatty acids, especially butyrate protects kidney from ischemia/reperfusion injury. J. Invest. Med..

[48] Nicolas G.R., Chang P.V. (2019). Deciphering the Chemical Lexicon of Host-Gut Microbiota Interactions. Trends Pharmacol. Sci..

[49] Hawkes C.G., Hinson A.N., Vashishta A., Read C.B., Carlyon J.A., Lamont R.J., Uriarte S.M., Miller D.P. (2023). *Selenomonas sputigena* Interactions with Gingival Epithelial Cells That Promote Inflammation. Infect. Immun..

[50] Pomeroy C., Shanholtzer C.J., Peterson L.R. (1987). Selenomonas bacteraemia—Case report and review of the literature. J. Infect..

[51] Pratama R., Schneider D., Böer T., Daniel R. (2019). First Insights into Bacterial Gastrointestinal Tract Communities of the Eurasian Beaver (Castor fiber). Front. Microbiol..

[52] Bai Y., Zhou X., Li N., Zhao J., Ye H., Zhang S., Yang H., Pi Y., Tao S., Han D. (2021). In Vitro Fermentation Characteristics and Fiber-Degrading Enzyme Kinetics of Cellulose, Arabinoxylan, β-Glucan and Glucomannan by Pig Fecal Microbiota. Microorganisms.

[53] Schofield B.J., Lachner N., Le O.T., McNeill D.M., Dart P., Ouwerkerk D., Hugenholtz P., Klieve A.V. (2018). Beneficial changes in rumen bacterial community profile in sheep and dairy calves as a result of feeding the probiotic Bacillus amyloliquefaciens H57. J. Appl. Microbiol..

[54] Jiang L., Shang M., Yu S., Liu Y., Zhang H., Zhou Y., Wang M., Wang T., Li H., Liu Z. (2022). A high-fiber diet synergizes with Prevotella copri and exacerbates rheumatoid arthritis. Cell. Mol. Immunol..

[55] Zhao Y., Xie B., Gao J., Zhao G. (2020). Dietary Supplementation with Sodium Sulfate Improves Rumen Fermentation, Fiber Digestibility, and the Plasma Metabolome through Modulation of Rumen Bacterial Communities in Steers. Appl. Environ. Microbiol..

[56] Wang C.S., Li W.B., Wang H.Y., Ma Y.M., Zhao X.H., Yang H., Qian J.M., Li J.N. (2018). VSL#3 can prevent ulcerative colitis-associated carcinogenesis in mice. World J. Gastroenterol..

[57] Wang C., Li W., Wang H., Ma Y., Zhao X., Zhang X., Yang H., Qian J., Li J. (2019). Saccharomyces boulardii alleviates ulcerative colitis carcinogenesis in mice by reducing TNF-alpha and IL-6 levels and functions and by rebalancing intestinal microbiota. BMC Microbiol..

[58] Redding L.E., Berry A.S., Indugu N., Huang E., Beiting D.P., Pitta D. (2021). Gut microbiota features associated with Clostridioides difficile colonization in dairy calves. PLoS ONE.

[59] Schoeler M., Caesar R. (2019). Dietary lipids, gut microbiota and lipid metabolism. Rev. Endocr. Metab. Disord..

[60] Zhang X., Hou Z., Tian X., Wu D., Dai Q. (2022). Multi-omics reveals host metabolism associated with the gut microbiota composition in mice with dietary epsilon-polylysine. Food Funct..

[61] Tang Z., Song B., Zheng C., Zheng J., Yin Y., Chen J. (2021). Dietary Beta-Hydroxy-Beta-Methyl Butyrate Supplementation Affects Growth, Carcass Characteristics, Meat Quality, and Serum Metabolomics Profile in Broiler Chickens. Front. Physiol..

[62] Elango R. (2023). Tolerable Upper Intake Level for Individual Amino Acids in Humans: A Narrative Review of Recent Clinical Studies. Adv. Nutr..

[63] Jiao M., He W., Ouyang Z., Shi Q., Wen Y. (2022). Progress in structural and functional study of the bacterial phenylacetic acid catabolic pathway, its role in pathogenicity and antibiotic resistance. Front. Microbiol..

[64] Zhang B., Jiang M., Zhao J., Song Y., Du W., Shi J. (2022). The Mechanism Underlying the Influence of Indole-3-Propionic Acid: A Relevance to Metabolic Disorders. Front. Endocrinol..

[65] Agus A., Clement K., Sokol H. (2021). Gut microbiota-derived metabolites as central regulators in metabolic disorders. Gut.

[66] Gu X., Tohme R., Tomlinson B., Sakre N., Hasipek M., Durkin L., Schuerger C., Grabowski D., Zidan A.M., Radivoyevitch T. (2021). Decitabine- and 5-azacytidine resistance emerges from adaptive responses of the pyrimidine metabolism network. Leukemia.

[67] Devi N., Kaur K., Biharee A., Jaitak V. (2021). Recent Development in Indole Derivatives as Anticancer Agent: A Mechanistic Approach. Anti-Cancer Agents Med. Chem..

[68] Cansev M., Watkins C.J., van der Beek E.M., Wurtman R.J. (2005). Oral uridine-5’-monophosphate (UMP) increases brain CDP-choline levels in gerbils. Brain Res..

[69] Wang L., Albrecht M.A., Wurtman R.J. (2007). Dietary supplementation with uridine-5′-monophosphate (UMP), a membrane phosphatide precursor, increases acetylcholine level and release in striatum of aged rat. Brain Res..

[70] Martin S.A., Brash A.R., Murphy R.C. (2016). The discovery and early structural studies of arachidonic acid. J. Lipid. Res..

[71] Szczuko M., Kikut J., Komorniak N., Bilicki J., Celewicz Z., Zietek M. (2020). The Role of Arachidonic and Linoleic Acid Derivatives in Pathological Pregnancies and the Human Reproduction Process. Int. J. Mol. Sci..

[72] Cheng Q., Tian L., Liang H., Luo Y. (2019). Research progress of 12-HETE in the inflammation and oxidative stress. Zhonghua Wei Zhong Bing Ji Jiu Yi Xue.

[73] Gagnon K.J., Lefort N., Poirier S.J., Barnett D.A., Surette M.E. (2018). 5-lipoxygenase-dependent biosynthesis of novel 20:4 n-3 metabolites with anti-inflammatory activity. Prostaglandins Leukot. Essent. Fat. Acids.

[74] Hernandez-Patlan D., Solis-Cruz B., Latorre J.D., Merino-Guzman R., Morales R.M., Ausland C., Hernandez-Velasco X., Ortiz H.O., Delgado R., Hargis B.M. (2022). Whole-Genome Sequence and Interaction Analysis in the Production of Six Enzymes From the Three Bacillus Strains Present in a Commercial Direct-Fed Microbial (Norum) Using a Bliss Independence Test. Front. Vet. Sci..

[75] Omar A.E., Al-Khalaifah H.S., Ismail T.A., Abd E.R., El-Mandrawy S., Shalaby S.I., Ibrahim D. (2021). Performance, Serum Biochemical and Immunological Parameters, and Digestive Enzyme and Intestinal Barrier-Related Gene Expression of Broiler Chickens Fed Fermented Fava Bean By-Products as a Substitute for Conventional Feed. Front. Vet. Sci..

[76] Zuo Z.H., Shang B.J., Shao Y.C., Li W.Y., Sun J.S. (2019). Screening of intestinal probiotics and the effects of feeding probiotics on the growth, immune, digestive enzyme activity and intestinal flora of Litopenaeus vannamei. Fish Shellfish Immunol..

