# Supplementary material for: Probiotic-Fermented Distillers Grain Alters the Rumen Microbiome, Metabolome, and Enzyme Activity, Enhancing the Immune Status of Finishing Cattle
Source: Animals (Basel). 2023 Dec 7;13(24):3774. doi: 10.3390/ani13243774 (PMC10740804; doi:10.3390/ani13243774)
Supplement: Supplementary file 1 [file animals-13-03774-s001.zip › Figure S1.pdf]

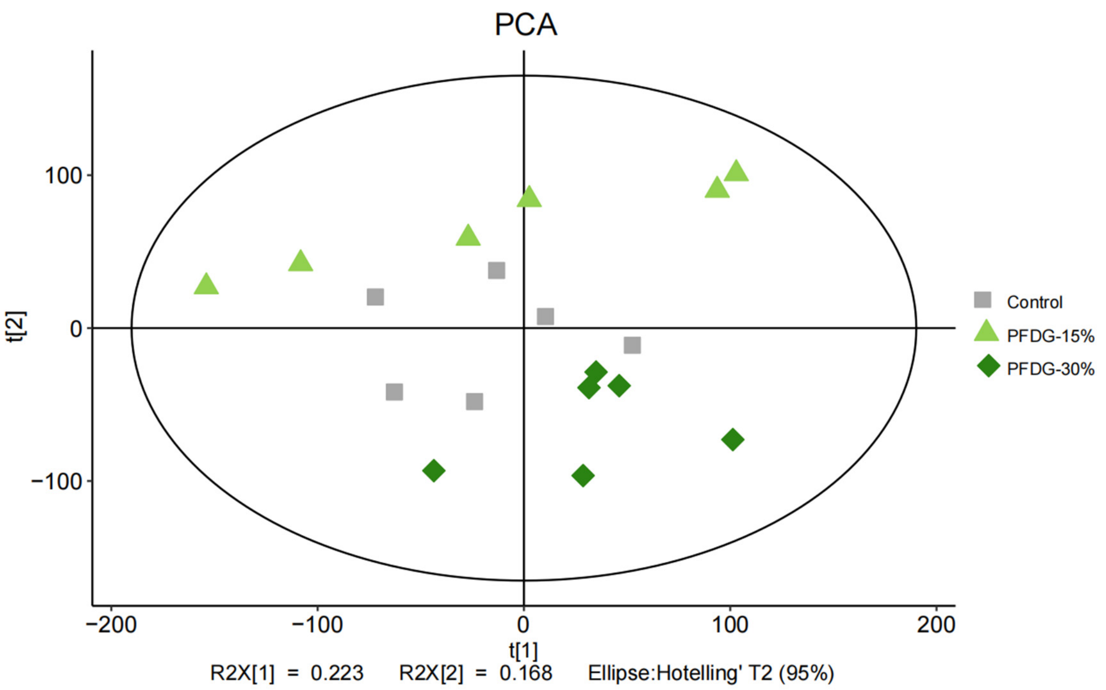

Figure S1. PCA score plots generated from rumen fluid metabolic profiles. Control, PFDG-15% and PFDG-30% represent the group without PEDG supplementation, the group with 15% PFDG substituting 15% concentrate, and the group with 30% PFDG substituting 30% concentrate, respectively (*n*=6).
