# Supplementary material for: Probiotic-Fermented Distillers Grain Alters the Rumen Microbiome, Metabolome, and Enzyme Activity, Enhancing the Immune Status of Finishing Cattle
Source: Animals (Basel). 2023 Dec 7;13(24):3774. doi: 10.3390/ani13243774 (PMC10740804; doi:10.3390/ani13243774)
Supplement: Supplementary file 1 [file animals-13-03774-s001.zip › Table S1-S2.pdf]

Table S1 Information on differential metabolites in the Control group vs. PFDG-15% group[1].

Differential Metabolites	Q-value[2]	VIP[3]	FC[4]	*P*
12-Hydroxystearic acid	0.317	12.165	1.714	0.048
PA(8:0/i-18:0)	0.265	10.173	0.702	0.033
Octadecanedioic acid	0.002	9.396	3.044	0.000
4α-formyl-4β-methyl-5α-cholesta-8,24-dien-3β-ol	0.000	9.324	0.177	0.000
D-Fructose	0.318	9.032	0.365	0.049
7-Methyl-2-(2-furyl)-1,8-naphthyridine-4(1H)-one	0.069	8.114	1.596	0.004
PC(16:0/18:2(9E,11E))	0.008	7.470	3.258	0.000
PE(P-16:0/15:0)	0.001	7.311	2.014	0.000
L-Alloisoleucine	0.124	7.188	0.479	0.009
N-(15-methyl-3-(13-methyl-4Z-tetradecenoyloxy)-hexadecanoyl)-glycine methyl ester	0.286	6.938	1.867	0.039
Glyzarin	0.258	6.888	1.096	0.032
2-Hydroxycinnamic acid	0.158	6.852	0.569	0.014
9S,11R,15S-trihydroxy-2,3-dinor-13E-prostaenoic acid-cyclo[8S,12R]	0.133	6.671	1.351	0.010
Barbital	0.000	6.560	4.112	0.000
5-Thymidylic acid	0.242	6.083	0.659	0.028
4-(D-Glucopyranosyloxy)-4,4'-diaponeurosporene	0.195	5.812	0.621	0.020
Berkeleylactone F	0.001	5.719	1.677	0.000
2-C-methyl-D-erythritol-4-phosphate	0.216	5.670	0.457	0.023
N-Acetylmannosamine	0.207	5.532	0.592	0.022
12(S),20-DiHETE	0.002	5.362	3.535	0.000
1-Nitro-3,5-dinitroso-1,3,5-triazinane	0.289	5.173	0.423	0.039
12S-hydroxy-16-heptadecynoic acid	0.034	5.062	1.583	0.002
Diethylene glycol dimethacrylate	0.014	5.035	0.696	0.001
1-O-(2R-methoxy-hexadecyl)-sn-glycerol	0.153	4.964	1.082	0.013
Uridine 5'-monophosphate	0.280	4.936	0.748	0.037
(3R,7R)-1,3,7-Octanetriol	0.151	4.932	1.111	0.013
N-Acetylmuramate	0.322	4.907	0.448	0.050
Palmitoylethanolamide	0.204	4.813	0.462	0.021
5-Aminopentanoic acid	0.271	4.784	0.693	0.035
Metharbital	0.000	4.695	2.365	0.000
Benzofuran, 4,7-dimethyl-	0.001	4.694	2.375	0.000
PS(22:0/0:0)	0.242	4.658	0.684	0.028
Indoleacrylic acid	0.047	4.606	0.594	0.002
4,4-Dimethylcholesta-8,14,24-trienol	0.001	4.548	0.238	0.000
1-(2-methoxy-17Z-tetracosenyl)-sn-glycero-3-phosphoserine	0.255	4.320	0.686	0.031
Hydroxy Tyrosol -Acetate	0.019	4.307	0.315	0.001
6-Hydroxypentadecanedioic acid	0.000	4.279	14.180	0.000
Phenol A	0.014	4.267	0.707	0.001
Methoxybrassenin A	0.156	4.257	1.180	0.014
Isoimperatorin	0.224	4.225	1.159	0.025

Pennelliiside C	0.010	4.129	10.565	0.000
Oleoylethanolamide	0.206	4.094	0.453	0.021
Phytic acid	0.313	3.952	1.233	0.047
ascr#24	0.000	3.820	17.527	0.000
1-((4-Methylsulfonyl)phenyl)-3-trifluoromethyl-5-(4-fluorophenyl)pyrazole	0.315	3.765	0.512	0.048
3-amino-2-naphthoic acid	0.001	3.605	1.852	0.000
2,6-Diamino-4-hydroxy-5-N-methylformamidopyrimidine	0.000	3.529	10.367	0.000
Gly-arg-gly-asp-ser	0.002	3.452	3.538	0.000
1-Galactopyranosyl-5-fluorouracil	0.152	3.355	1.165	0.013
3-(4-Hydroxyphenyl)-3,5,6,8-tetrahydro-2H-chromene-4,7-dione	0.089	3.343	1.256	0.006
N-Acetylproline	0.066	3.331	1.968	0.004
Hexadecanedioic acid	0.001	3.326	2.564	0.000
Atenolol	0.000	3.264	10.957	0.000
gamma-Glutamylproline	0.006	3.255	2.288	0.000
Creatinine nitrite nitrate	0.302	3.241	0.330	0.043
Diphenylamine	0.022	3.231	2.640	0.001
3,6,9,12-Tetraazatetradecanedioic acid, 3,6,9,12-tetrakis(carboxymethyl)-	0.001	3.228	3.601	0.000
Aspartyl-Cysteine	0.236	3.205	0.469	0.027
(+)-Isopilocarpine	0.000	3.205	11.001	0.000
(S)-3'-Hydroxy-4,4'-dimethoxydalbergione	0.192	3.193	1.167	0.019
4-(4-Hydroxyphenyl)-2-butanone O-[2,6-digalloylglucoside]	0.002	3.193	3.597	0.000
Methyl 3-[(4-anilino-2-methoxyphenyl)sulfamoyl]thiophene-2-carboxylate	0.299	3.174	1.142	0.042
Sodium phosphate (Na(H2PO4))	0.302	3.161	1.480	0.043
Flazine	0.000	3.131	15.043	0.000
Yangonin	0.268	3.123	1.181	0.034
4-amino-6,7-dihydro-5H-cyclopenta[b]pyridin-2-yl 4-methylbenzenesulfonate	0.002	3.118	3.767	0.000
6-Keto-prostaglandin F1a	0.287	3.091	0.706	0.039
Chiniofon	0.188	3.027	1.705	0.019
N-Benzoylaspartic acid	0.000	3.008	6.274	0.000
Benzofuran	0.138	2.989	0.548	0.011
PA(P-16:0/17:1(9Z))	0.004	2.976	2.196	0.000
Hydroxyprolylhydroxyproline	0.001	2.944	2.117	0.000
Morph	0.001	2.944	2.245	0.000
1-O-(2R-hydroxy-pentadecyl)-sn-glycerol	0.208	2.940	1.155	0.022
3-Hydroxy-2-oxoindole	0.000	2.886	5.985	0.000
Posaconazole	0.281	2.838	0.700	0.038
Cohibin C	0.163	2.811	8.184	0.015
5,6-Dihydro-11-methoxyyangonin	0.045	2.802	1.244	0.002
Infectocaryone	0.000	2.795	14.614	0.000
Haematopodin	0.156	2.793	1.666	0.013
Lamivudine-monophosphate	0.127	2.792	0.575	0.010
Arginyl-prolyl-proline	0.052	2.792	1.647	0.003
N-Succinyl-L,L-2,6-diaminopimelate	0.000	2.779	7.061	0.000
Sordarin	0.051	2.764	1.910	0.003

Indolepyruvate	0.000	2.712	20.393	0.000
Isomugineic acid	0.019	2.678	0.712	0.001
Lufenuron	0.293	2.668	0.710	0.040
dCMP	0.158	2.668	0.781	0.014
8-undecynoic acid	0.295	2.657	1.208	0.041
Polypropylene	0.273	2.645	0.502	0.035
Phaseolic acid	0.106	2.623	0.707	0.008
oscr#23	0.000	2.607	54.125	0.000
Manassantin B	0.080	2.605	0.460	0.005
3-O-Galloyl-1,4-galactarolactone	0.278	2.577	0.693	0.037
DG(20:4(5Z,7E,11Z,14Z)-OH(9)/0:0/i-12:0)	0.087	2.571	0.616	0.006
3-Methyl sulfolene	0.106	2.562	0.453	0.008
Tambulin 3,5-diacetate	0.157	2.546	1.194	0.014
22:3(5Z,9Z,19Z)(13Me,17Me,21Me)	0.177	2.541	0.809	0.017
DG(20:3(8Z,11Z,14Z)-2OH(5,6)/0:0/a-15:0)	0.274	2.514	3.039	0.035
3-oxo-tetracosanoic acid	0.019	2.484	18.045	0.001
Telbivudine	0.056	2.452	2.187	0.003
1-Benzenesulfonyl-5-ethyl-5-phenylhydantoin	0.238	2.414	0.743	0.027
4-Hydroxy-3-methoxy-2,10-bisaboladien-9-one	0.002	2.407	1.537	0.000
LysoPE(18:2(9Z,12Z)/0:0)	0.284	2.395	0.449	0.038
Methoxyfenozide	0.013	2.364	0.404	0.000
alpha,beta-Methylene ATP	0.023	2.333	0.483	0.001
L-Glutamic acid, N-((((1S)-1-carboxy-5-((((4-iodophenyl)amino)carbonyl)amino)pentyl)amino)carbonyl)-	0.310	2.331	0.403	0.045
Glycolic acid	0.316	2.328	0.596	0.048
3-Azido-7-hydroxycoumarin	0.247	2.323	1.140	0.029
PA(13:0/15:1(9Z))	0.177	2.317	0.594	0.017
Byssochlamic acid	0.232	2.315	1.125	0.026
Gibberellin A53	0.010	2.305	1.431	0.000
Ibudilast	0.140	2.293	0.402	0.011
D-Fucose	0.021	2.291	32.912	0.001
(S)-N-(4,5-Dihydro-1-methyl-4-oxo-1H-imidazol-2-yl)alanine	0.002	2.278	2.904	0.000
Toxin T2 tetrol	0.000	2.262	3.819	0.000
S-4-Hydroxymephenytoin	0.001	2.260	1.915	0.000
Deoxycholic acid	0.232	2.251	0.535	0.026
1-(6-Hydroxy-2-azabicyclo[2.2.1]heptane-3-carbonyl)pyrrolidine-2-carbonitrile	0.099	2.250	1.151	0.007
5-Nitroxystearic acid	0.162	2.246	7.356	0.015
Glycyl-l-histidyl-l-lysine	0.002	2.233	2.095	0.000
1-Hydroxyvitamin D5	0.036	2.203	0.453	0.002
Sarcoehrendin D	0.115	2.194	2.751	0.009
N-Succinyl-2-amino-6-ketopimelate	0.314	2.188	0.536	0.047
Riddelliine	0.000	2.181	15.837	0.000

Ascorbic acid 3-sulfate	0.312	2.175	0.722	0.047
Soyasapogenol C	0.168	2.160	0.076	0.015
2-Acetylaminophenoxazin-3-one	0.000	2.159	2.525	0.000
Ceratiolin	0.292	2.158	1.233	0.040
Ascorbyl palmitate	0.003	2.151	3.329	0.000
Myricatin	0.127	2.146	1.160	0.010
5'-Deoxy-5'-fluorouridine	0.000	2.139	10.360	0.000
Penicillin V	0.008	2.134	0.686	0.000
Aldosine	0.000	2.128	12.698	0.000
(+)-cis-abscisic aldehyde	0.000	2.123	2.339	0.000
PPA(16:0/18:1(9Z))	0.306	2.118	2.391	0.044
N-(7Z-tetradecenoyl)-homoserine lactone	0.111	2.113	1.104	0.008
4-Oxo-1-(3-pyridyl)-1-butanone	0.000	2.100	4.965	0.000
LysoPA(18:0/0:0)	0.090	2.094	1.208	0.006
Catechin 7,3'-di-O-gallate	0.254	2.085	0.411	0.031
Isoflupredone acetate	0.261	2.067	1.871	0.032
PS(P-20:0/21:0)	0.195	2.058	0.645	0.020
Cytosine	0.233	2.057	0.723	0.026
3'-Hydroxyamobarbital	0.008	2.055	2.180	0.000
Caffeinol	0.000	2.047	41.020	0.000
4-amino-4-deoxychorismate	0.001	2.047	2.949	0.000
Enterolactone 3'-sulfate	0.280	2.043	0.764	0.037
4alpha-formyl-ergosta-7,24(241)-dien-3beta-ol	0.109	2.035	0.703	0.008
Proflavine	0.036	2.033	1.770	0.002
Nordihydrocapsiate	0.068	2.032	1.158	0.004
oscr#21	0.218	2.024	0.679	0.024
Coumarin 102	0.000	2.001	4.096	0.000
7-Hydroxyoctanoylcarnitine	0.242	2.000	1.076	0.028
6-hydroxyoct-4-enoylglycine	0.000	1.993	7.213	0.000
Glutamylisoleucine	0.018	1.990	0.668	0.001
Alanylhydroxyproline	0.002	1.984	2.197	0.000
Doxercalciferol	0.165	1.974	0.701	0.015
3-(2-Hydroxyphenyl)propanoic acid	0.193	1.972	1.330	0.019
3alpha-Hydroxy-5beta-chola-7,9(11)-dien-24-oic Acid	0.142	1.969	0.456	0.012
4-Hydroxy-4-(3-pyridyl)-butanoic acid	0.005	1.951	1.762	0.000
Hydroxyprolyl-Glutamine	0.002	1.950	1.901	0.000
Cer(d16:2(4E,6E)/22:0)	0.321	1.947	2.021	0.049
N-(1-Hydroxyethyl)octadecanamide	0.001	1.945	1.768	0.000
Butyrolactone derivative	0.010	1.944	2.268	0.000
7-Hydroxyflumequine	0.208	1.936	0.627	0.022
Isoleucylproline	0.000	1.932	4.819	0.000
2-Phenylaminoadenosine	0.001	1.920	4.032	0.000
2,6-Diaminopurine 2',3'-dideoxyriboside	0.207	1.918	0.833	0.022
N-(4-Amino-5-methoxy-2-methylphenyl)benzamide	0.000	1.909	112.05	0.000

			0	
6-Hydroxymelatonin	0.000	1.907	2.758	0.000
Iloperidone	0.002	1.907	3.406	0.000
Uracil	0.266	1.902	0.622	0.034
Caffeic acid 3-O-glucuronide	0.086	1.899	0.604	0.006
(2s)-2-[6-(Sulfooxy)naphthalen-2-yl]propanoic acid	0.019	1.895	0.692	0.001
2-({6-Amino-3,5-dicyano-4-[4-(cyclopropylmethoxy)phenyl]pyridin-2-yl}sulfanyl)acetamide	0.285	1.886	0.502	0.038
12-oxo-PDA	0.065	1.884	1.259	0.004
5-(2-Aminopropyl)benzofuran	0.027	1.858	1.820	0.001
2,3-Bis(4-hydroxyphenyl)propionitrile	0.002	1.856	4.221	0.000
D-Xylonate	0.000	1.856	1.837	0.000
VULPINIC ACID	0.256	1.845	1.200	0.031
Neamine	0.003	1.844	533325448.192	0.000
Polyglycerol esters of fatty acids	0.003	1.811	234.647	0.000
Guanidinosuccinic acid	0.165	1.808	0.429	0.015
Urocanic acid	0.134	1.805	0.570	0.011
Isoleucyl-Glutamate	0.248	1.799	0.707	0.029
((2-Amino-3-((2-amino-3-((carboxymethyl)amino)-3-oxopropyl)dithio)propanoyl)amino)acetic acid	0.005	1.795	0.679	0.000
5,6-Methylenedioxy-2-aminoindane	0.031	1.793	2.888	0.001
1-(6-[3]-ladderane-hexanoyl)-2-(8-[3]-ladderane-octanyl)-sn-glycero-3-phospho-(1'-sn-glycerol)	0.170	1.788	9.540	0.016
1-(8-[5]-ladderane-octanyl)-2-(8-[3]-ladderane-octanyl)-sn-glycero-3-phospho-(1'-sn-glycerol)	0.081	1.782	0.739	0.005
L-DOPA n-Butyl Ester	0.000	1.782	8.084	0.000
Talaromycin A	0.215	1.780	0.737	0.023
Tyrosyl-Proline	0.149	1.779	0.544	0.012
Fenitrothion	0.174	1.778	0.550	0.016
Indigo Carmine	0.000	1.771	3.791	0.000
4,1-Benzoxazepine	0.157	1.767	0.539	0.014
Histidinyl-Lysine	0.002	1.766	5.979	0.000
TRIBUTYL PHOSPHATE	0.164	1.762	1.368	0.015
PE(TXB2/18:0)	0.028	1.760	0.595	0.001
(2E,8E)-10-hydroxydeca-2,8-dien-4-ynoylcarnitine	0.004	1.757	4.083	0.000
N-oleoyl glutamine	0.000	1.754	4.001	0.000
Methyl-3H-thymidine	0.012	1.753	2.868	0.000
1-(2-Hydroxyethoxy)methyl-5-methyluracil	0.000	1.750	6.570	0.000
Termitomycesphin E	0.062	1.747	1.668	0.004
11-nitro-1-undecene	0.128	1.746	1.109	0.010
L-Pyridosine	0.251	1.742	0.597	0.030

3-Ethylheptadecan-2-one	0.074	1.734	0.667	0.005
HARMOL	0.122	1.733	1.543	0.009
Formylfusarochromanone	0.000	1.722	3.822	0.000
10-Deoxymethynolide	0.044	1.706	1.363	0.002
DG(22:5(4Z,7Z,10Z,13Z,19Z)-O(16,17)/0:0/i-13:0)	0.134	1.704	0.665	0.011
Amantadine	0.311	1.703	1.822	0.046
Glucoiberin	0.077	1.688	0.641	0.005
Norketobemidone	0.000	1.687	2.705	0.000
3-(Aminomethyl)-2,5,9-trimethyl-7H-furo[3,2-g]chromen-7-one	0.001	1.680	46.244	0.000
Aegelinol	0.273	1.677	1.198	0.035
2-(5-Methoxy-1H-indol-3-yl)ethyl acetate	0.000	1.676	3.740	0.000
Stearoyllactic acid	0.006	1.675	12.494	0.000
Cerulenin	0.000	1.670	5.834	0.000
Ergosine	0.141	1.668	3.344	0.012
HDOPA	0.255	1.667	0.669	0.031
JWH 073 N-butanoic acid metabolite-d5	0.320	1.664	0.622	0.049
2-Cyanohept-2-enoic acid	0.000	1.660	6.246	0.000
Picolinic acid	0.303	1.660	0.728	0.043
PPA(18:1(9Z)/18:1(9Z))	0.091	1.654	11.893	0.006
Prolylproline	0.134	1.653	1.467	0.011
Tamsulosin	0.002	1.651	2.292	0.000
6beta-Hydroxycampest-4-ene-3-one	0.190	1.640	0.761	0.019
2-[[5-[3-(Dimethylamino)propyl]-2-methylpyridin-3-yl]amino]-9-(trifluoromethyl)-5,7-dihydropyrimido[5,4-d][1]benzazepine-6-thione	0.043	1.638	1.775	0.002
Non-sulfonylurea	0.000	1.638	3.122	0.000
Phenethylamine glucuronide	0.000	1.633	3.174	0.000
DG(TXB2/0:0/17:0)	0.243	1.619	0.627	0.028
thioinosine monophosphate	0.052	1.619	0.711	0.003
N-palmitoyl threonine	0.022	1.618	2.572	0.001
Piroxantrone	0.001	1.611	4.720	0.000
1-Methylcytosine	0.309	1.602	1.218	0.045
PC(18:2(10E,12Z)+=O(9)/17:0)	0.047	1.600	1.415	0.003
Porphobilinogen	0.003	1.599	3.573	0.000
Met-Gln-Cys-Asn-Ser	0.063	1.598	3.207	0.004
N1-Acetylspermidine	0.041	1.592	0.571	0.002
Indolelactic acid	0.293	1.589	0.226	0.041
PA(a-21:0/10:0)	0.221	1.587	14.803	0.024
FS4 toxin	0.250	1.586	0.823	0.030
Metoprolol acid	0.000	1.578	22.747	0.000
Tetradecanoylcarnitine	0.000	1.576	23.649	0.000
L-Phenylalanine	0.062	1.575	0.481	0.004
DG(10:0/0:0/8:0)	0.027	1.571	2.173	0.001
Gentiatibetine	0.020	1.565	2.932	0.001
5'-Amino-5'-deoxyadenosine	0.108	1.564	0.694	0.008

Hydrocortisone cypionate	0.162	1.561	0.687	0.015
Gomphrenin II	0.317	1.560	1.302	0.048
3,4-Dihydroxybenzaldehyde	0.171	1.557	1.224	0.016
5-Phosphoribosylamine	0.280	1.549	0.578	0.037
2-Methylhippuric acid	0.004	1.547	0.436	0.000
4-Bis(2-hydroxyethyl)amino-L-phenylalanine	0.000	1.540	3.118	0.000
Dehydroxymethylflazine	0.005	1.538	23.497	0.000
O-Desmethylangolensin	0.174	1.534	0.363	0.016
Cbmida	0.196	1.534	1.184	0.020
2-Amino-5-phenylpyridine	0.000	1.534	1.899	0.000
valine lactate	0.000	1.534	6.916	0.000
N6,N6,N6-Trimethyl-L-lysine	0.143	1.531	0.483	0.012
2-(2-Aminopropanoylamino)bicyclo[3.1.0]hexane-2,6-dicarboxylic acid	0.013	1.527	0.628	0.000
Carazolol	0.000	1.525	5.032	0.000
Altamisic acid; 3-[(3aS,8R,8aR)-8-Hydroxy-6,8-dimethyl-3-methylene-2-oxo-3,3a,4,5,8,8a-hexahydro-2H-cyclohepta[b]furan-7-yl]propanoic acid	0.219	1.522	0.793	0.024
Droxidopa	0.188	1.521	1.286	0.019
2-Acetamido-2,6-dideoxygalactose	0.057	1.511	1.190	0.003
(2R,7''S)-8-(1-phenyl-2-carboxyethyl)pinocembrin	0.011	1.510	1.925	0.000
3,5-Pyridinedicarboxylic acid, 1,4-dihydro-2,4,6-trimethyl-, diethyl ester	0.000	1.506	50.668	0.000
Galaxolide	0.260	1.500	1.076	0.032
Pseudoyohimbine	0.011	1.498	0.467	0.000
Tumonoic Acid A	0.213	1.491	2.020	0.023
3-Methoxyanthranilate	0.000	1.488	2.913	0.000
Xanthyletin	0.000	1.481	2.272	0.000
3-(2-(Benzoxazol-2-yl)ethyl)-5-ethyl-6-methylpyridin-2(1H)-one	0.285	1.480	0.625	0.038
Alpha-Linolenoyl ethanolamide	0.065	1.478	0.336	0.004
DG(a-15:0/22:6(4Z,8Z,10Z,13Z,16Z,19Z)-OH(7)/0:0)	0.251	1.478	0.664	0.030
1-O-Pentylglycerol	0.205	1.465	1.125	0.021
Tripropyl phosphate	0.003	1.456	1.759	0.000
(1R,6R)-6-hydroxy-2-succinylcyclohexa-2,4-diene-1-carboxylate	0.013	1.447	0.497	0.000
Glutamylleucine	0.232	1.438	0.790	0.026
Perindopril	0.007	1.431	0.372	0.000
D-Arginine	0.067	1.429	0.612	0.004
1,1'-(1,8-Dioxo-1,8-octanediyl)bis-2,5-pyrrolidinedione	0.000	1.429	3.601	0.000
Kynurenic acid	0.205	1.428	0.633	0.021
Fostemsavir	0.203	1.426	0.325	0.021
Thr-Leu	0.000	1.425	8.869	0.000
Fucose 1-phosphate	0.003	1.425	0.482	0.000
N-acetyl-2-carboxy Benzenesulfonamide	0.212	1.425	0.625	0.022
Tezacaftor	0.116	1.423	1.380	0.009
3-Indolebutyric acid	0.029	1.422	0.479	0.001
Ketobemidone	0.000	1.420	21.596	0.000

12-Oxo-2,3-dinor-10,15-phytodienoic acid	0.002	1.420	1.571	0.000
Eltoprazine	0.014	1.419	2.223	0.001
1alpha,25-dihydroxy-24-oxo-23-azavitamin D2 / 1alpha,25-dihydroxy-24-oxo-23-azaergocalciferol	0.210	1.414	0.740	0.022
Glycyl-Phenylalanine	0.156	1.412	0.592	0.014
Calcitriol	0.006	1.409	18.247	0.000
Stavudine triphosphate	0.230	1.409	0.333	0.026
Petroformynic acid B	0.216	1.406	4.275	0.023
1-Deoxy-1-(N6-lysino)-D-fructose	0.093	1.406	0.197	0.006
S-Oxide	0.181	1.402	1.094	0.017
beta-D-ribosylnicotinate	0.004	1.400	3.335	0.000
Indecainide	0.001	1.395	0.345	0.000
Mefloquine	0.278	1.394	0.610	0.037
1-(beta-D-Ribofuranosyl)-1,4-dihydronicotinamide	0.002	1.394	5.779	0.000
7-Methylinosine	0.000	1.387	13.627	0.000
Dodeca-3,5,7-trienoylcarnitine	0.308	1.386	0.644	0.045
5-O-Methylleridol	0.000	1.384	2.300	0.000
Abscisic alcohol	0.216	1.383	0.896	0.023
Dyphylline	0.000	1.379	22.508	0.000
Indirubin-3'-monoxime	0.071	1.375	1.815	0.004
gamma-Glutamyl-S-methylcysteine sulfoxide	0.204	1.367	1.247	0.021
6-Methoxymellein	0.001	1.362	4.093	0.000
Cholic acid	0.190	1.355	0.460	0.019
2-Hydroxy-4-[(1R)-1-hydroxy-8-methyl-6-oxononyl]-3-methyl-2H-furan-5-one	0.256	1.343	0.857	0.031
1,9-Heptadecadiene-4,6-diyn-3-ol, (3R,9Z)-	0.126	1.340	1.414	0.010
(10S,11S)-Pterosin C	0.000	1.339	2.426	0.000
Isolubimin	0.001	1.338	1.293	0.000
6-Methylthioinosine	0.318	1.337	0.578	0.049
[6]-Dehydrogingerdione	0.135	1.336	0.821	0.011
Methylene bisacrylamide	0.079	1.335	2.585	0.005
11-Hydroxyyohimbine	0.000	1.335	0.413	0.000
Serylvaline	0.061	1.334	0.596	0.003
Campesteryl glucoside	0.038	1.331	0.515	0.002
10E-Pentadecen-6,8-diynoic acid	0.041	1.327	0.818	0.002
Cer(d18:2(4E,14Z)/20:5(7Z,9Z,11E,13E,17Z)-3OH(5,6,15))	0.166	1.326	0.499	0.015
Acetamide, 2-(diethylamino)-N-(2-(octyloxy)phenyl)-	0.003	1.326	3.987	0.000
5'-Phosphoribosyl-N-formylglycinamide	0.000	1.325	18.211	0.000
L-Norleucine	0.035	1.322	1.470	0.002
PA(22:6(5Z,8E,10Z,13Z,15E,19Z)-2OH(7S, 17S)/10:0)	0.031	1.318	0.412	0.001
Manoalide	0.002	1.317	3.366	0.000
lysine phosphoester	0.069	1.316	0.640	0.004
ascr#18	0.086	1.315	1.268	0.006
19-Hydroxy-10S,19-dihydrovitamin D3	0.066	1.309	0.716	0.004

4alpha-Hydroxymethyl-4beta-methyl-5alpha-cholesta-8,24-dien-3beta-ol	0.002	1.304	0.084	0.000
5-((Z)-nonadec-8-en-1-yl)resorcinol	0.077	1.304	0.616	0.005
Ehrensteroid F	0.267	1.298	1.966	0.034
trioxyethylene dimethacrylate	0.304	1.297	1.789	0.044
Melledonol	0.009	1.295	1.553	0.000
L-Homoserine	0.289	1.292	0.656	0.039
PGF1a alcohol	0.005	1.291	11.387	0.000
Proline glutamate	0.002	1.289	2.720	0.000
xi-2,3-Dihydro-2-oxo-1H-indole-3-acetic acid	0.221	1.285	0.622	0.024
Borapetoside	0.207	1.284	0.493	0.021
9-hydroxy-traumatin	0.160	1.283	0.880	0.014
ascr#28	0.175	1.283	1.259	0.016
6,2'4'-Trihydroxy-2-phenylbenzofuran	0.000	1.278	30.949	0.000
Sedoheptulose 1,7-bisphosphate	0.001	1.275	0.561	0.000
Proline betaine	0.162	1.273	1.282	0.014
(Z)-Tamarindienal	0.000	1.268	1.434	0.000
Triamcinolone	0.237	1.267	1.959	0.027
3-Methoxytyramine	0.002	1.266	2.232	0.000
Catechin 5,4'-di-O-beta-D-glucopyranoside	0.001	1.262	3.743	0.000
17-dimethylarsinoyl-9Z-heptadecenoic acid	0.039	1.260	0.551	0.002
2-Acetyl-6-methylpyridine	0.000	1.259	13.133	0.000
Tyrosyl-Asparagine	0.180	1.254	0.581	0.017
Glycylleucine	0.179	1.247	0.692	0.017
Cucurbic acid	0.267	1.246	0.830	0.034
Cis-stilbene oxide	0.003	1.245	1.901	0.000
PE-NMe2(18:3(6Z,9Z,12Z)/14:0)	0.178	1.245	7.389	0.017
DG(10:0/0:0/10:0)	0.008	1.244	257669438.872	0.000
3-Dehydrosphinganine	0.180	1.241	0.871	0.017
(Z)-15-Oxo-11-eicosenoic acid	0.045	1.240	3.569	0.002
Epoxyganoderiol C	0.217	1.239	0.071	0.024
Prolyl-Alanine	0.000	1.239	1.944	0.000
Ibuprofen glucuronide	0.302	1.239	0.628	0.043
8-Methoxy-6,7-methylenedioxycoumarin	0.002	1.239	3.754	0.000
Cyclocalopin D	0.068	1.234	2.844	0.004
3,11,12-Trihydroxy-1(10)-spirovetiven-2-one	0.157	1.230	0.868	0.014
(S)-[10]-Gingerol	0.000	1.222	17.239	0.000
Collimonin D	0.040	1.222	1.205	0.002
Leucyl-Gamma-glutamate	0.079	1.218	0.697	0.005
3R-hydroxy-5Z-dodecenoic acid	0.312	1.217	0.820	0.046
Epiheterodendrin	0.000	1.215	6.905	0.000
Stercobilinogen	0.111	1.210	0.370	0.008
tyrosine lactate	0.079	1.209	1.609	0.005

(+)-gamma-Hydroxy-L-homoarginine	0.001	1.203	0.190	0.000
Pyridoxal	0.003	1.202	1.476	0.000
Isamoxole	0.000	1.201	212495381.128	0.000
Nicametate	0.000	1.200	5.426	0.000
4-Methyl-1-phenyl-2-pentanone	0.102	1.200	1.074	0.007
5,2'-Dihydroxy-7-methoxy- 6,8-dimethylflavone	0.266	1.197	0.861	0.034
Deltoidin A	0.000	1.190	0.151	0.000
3-Acetoxystearic acid	0.245	1.187	1.506	0.029
N-[(2Z)-2-Methoxyiminopropyl]-7H-purin-6-amine	0.007	1.187	0.456	0.000
Metiazinic acid	0.005	1.184	26.891	0.000
Salacinol	0.312	1.180	0.558	0.047
Metomidate	0.158	1.177	2.020	0.014
ortho-Hydroxyphenylacetic acid	0.025	1.176	1.456	0.001
3S-hydroxy-4R-methyl-2S-(n-eicos-11?-yn-19?-enyl)butanolide	0.003	1.174	9.045	0.000
3,4-Bis(carboxymethyl)-3,4-dihydroxyhexanedioic acid	0.044	1.173	0.648	0.002
Chenodeoxycholylglutamic acid	0.049	1.168	0.539	0.003
4-(2-Naphthyloxy)-2-butyne-1-amine	0.000	1.167	143.343	0.000
3-[(2-Oxoacetyl)oxy]-4-(trimethylazaniumyl)butanoate	0.210	1.167	4.081	0.022
Adiponitrile	0.089	1.166	1.518	0.006
1-(3-Carboxypropylcarbamoyl)-5-fluorouracil	0.000	1.163	8.949	0.000
Oxprenolol	0.073	1.162	1.153	0.005
L-Aspartic acid	0.303	1.161	0.610	0.043
Olomoucine	0.000	1.161	158.669	0.000
L-Proline, 1-(1-L-leucyl-L-prolyl)-	0.220	1.159	0.375	0.024
19-hydroxy-Resolvin E1	0.186	1.157	0.627	0.018
Gorgostane-3beta,5alpha,6beta,11alpha,12beta-pentol 12-acetate	0.262	1.153	0.589	0.033
1-O-(2R-methoxy-4Z-docosenyl)-sn-glycerol	0.243	1.150	2.104	0.028
Pomalidomide	0.000	1.150	4.143	0.000
Isorhamnetin 3-O-beta-D-2",3",4"-triacetylglucopyranoside	0.213	1.149	1.372	0.023
Polypropylene glycol (m w 1,200-3,000)	0.100	1.148	1.232	0.007
2-Hydroxyquinoline-3-carboxylic acid	0.201	1.146	1.150	0.020
4-p-Coumaroyl-1,5-quinolactone	0.318	1.144	0.858	0.048
SERICETIN DIACETATE	0.215	1.144	2.007	0.023
Histidylprolineamide	0.172	1.144	0.530	0.016
Furoparadine	0.094	1.141	1.826	0.006
4-Hydroxyquinoline	0.202	1.140	0.656	0.021
Persenone B	0.033	1.140	17.675	0.002
6-Methyladenine	0.257	1.139	0.758	0.032
xymedon	0.000	1.139	6.393	0.000
N-alpha-Acetyl-L-citrulline	0.278	1.138	0.741	0.037

7-hydroxy-dodecanoic acid	0.028	1.138	157.618	0.001
(4-Ethylnaphthalen-1-yl)[1-(5-fluoropentyl)-1H-indol-3-yl]methanone	0.287	1.138	0.560	0.039
Diethyl 1,4-dihydro-2,6-dimethyl-3,5-pyridinedicarboxylate	0.000	1.135	3.827	0.000
Brassicanal A	0.024	1.135	0.698	0.001
Aspidospermatine	0.005	1.131	0.398	0.000
L-gamma-Glutamyl-beta-phenyl-beta-L-alanine	0.207	1.129	0.700	0.022
Cerevisterol	0.213	1.128	1.550	0.023
2-Butylbenzothiazole	0.212	1.127	0.842	0.023
1-(2,3-Dihydro-5,6-dimethyl-1H-pyrrolizin-7-yl)ethanone	0.000	1.126	94.887	0.000
Serylphenylalanine	0.090	1.123	1.296	0.006
(22E,24R)-15alpha-hydroxyergosta-4,6,8(14),22-tetraen-3-one	0.000	1.122	7.337	0.000
N-methyl-4,6,7-trihydroxy-1,2,3,4-tetrahydroisoquinoline	0.089	1.121	1.484	0.006
Cinnamaldehyde	0.000	1.114	2.405	0.000
Artabsinolide D	0.158	1.114	0.832	0.014
6-Butyryl-5-hydroxy-4-phenylseselin	0.001	1.114	2.291	0.000
Carnosol	0.191	1.114	1.299	0.019
Diphenylhydantoic acid	0.000	1.113	3.890	0.000
L-Thyronine	0.000	1.112	19.005	0.000
(4S,6R)-6-[(1E)-4,4-Bis(4-fluorophenyl)-3-(1-methyltetrazol-5-yl)buta-1,3-dienyl]-4-hydroxyoxan-2-one	0.012	1.111	5.174	0.000
Pretyrosine	0.062	1.109	0.723	0.004
Cerebroside E	0.254	1.104	0.584	0.031
4-Phenyl-3-buten-2-ol	0.094	1.102	1.369	0.006
N-Phenyl-p-phenylenediamine	0.000	1.098	3.575	0.000
3-Methyloxindole	0.303	1.094	0.620	0.043
L-Xylulose	0.124	1.094	0.542	0.009
(3beta,23E)-3-Hydroxy-27-norcycloart-23-en-25-one	0.275	1.093	0.265	0.036
cis-3-Hexenyl salicylate	0.031	1.092	1.195	0.001
4-Nitrophenyl beta-D-glucopyranoside	0.000	1.088	4.756	0.000
Glycylvaline	0.031	1.086	0.585	0.001
9-(1,3-Dioxolan-2-yl)purine-2,6-diamine	0.046	1.085	1.455	0.002
Cadabicilone	0.001	1.084	4.559	0.000
Acetylfentanyl	0.078	1.083	0.390	0.005
Glycylproline	0.276	1.081	1.251	0.036
13,14-dihydro-15-keto-PGE1	0.009	1.081	5.546	0.000
Isopropyl dodecylfluorophosphonate	0.066	1.079	2.065	0.004
7,2'-Dihydroxy-6,8-dimethyl-4',5'-methylenedioxyflavan	0.000	1.078	57.215	0.000
Mevalonic acid	0.003	1.078	2.134	0.000
DL-Ornithino-L-alanine	0.001	1.067	2.873	0.000
(3S,7S)-Jasmonic acid	0.114	1.066	0.801	0.008
L-Theanine	0.007	1.065	1.654	0.000
Procaine	0.060	1.065	1.457	0.003
Quadrangolin A;	0.033	1.064	0.863	0.002

2-[(1S,2S,4aR,8aS)-1-Hydroxy-4a-methyl-8-methylenedecahydro-2-naphthalenyl]acrylic acid				
4-(Trifluoromethyl)benzaldehyde	0.015	1.061	1.568	0.001
2-[(6-Aminopurin-9-yl)methoxy]ethyl dihydrogen phosphate	0.000	1.057	146.302	0.000
9-(4-Fluoro-3-(hydroxymethyl)butyl)guanine	0.312	1.055	0.503	0.047
4,6-O-Ethylidene-D-glucose	0.010	1.053	1.679	0.000
2-(2-Oxopropanoyloxy)butanedioic acid	0.039	1.052	0.631	0.002
Sibrafiban	0.093	1.049	1.847	0.006
Symmetric dimethylarginine	0.257	1.041	0.454	0.032
(2R,3S)-3-Hydroxy-1,1-dimethylpyrrolidin-1-ium-2-carboxylate	0.138	1.040	1.376	0.011
N-[4-[(6-Methoxypyrimidin-4-yl)sulfamoyl]phenyl]acetamide	0.237	1.036	2.206	0.027
Aspartyl-Leucine	0.100	1.035	0.785	0.007
Dattelic acid	0.174	1.034	1.643	0.016
2-Hydroxyethyl glucosinolate	0.242	1.031	0.526	0.028
Gyrophoric acid	0.280	1.030	1.162	0.037
Fasoracetam	0.000	1.029	90.941	0.000
Naphthalen-1-yloxy-oxido-oxophosphanium	0.138	1.027	0.753	0.011
(9S,10E,12S,13S)-9,12,13-Trihydroxy-10-octadecenoic acid	0.249	1.026	1.299	0.030
Niridazole	0.123	1.024	0.806	0.009
Tyrosyl-Glutamate	0.147	1.020	0.386	0.012
3-(Carboxymethyl)-3-hydroxypentanedioic acid	0.181	1.019	0.510	0.017
(2S,3R,4S,5R,6R)-6-Ethyloxane-2,3,4,5-tetrol	0.002	1.018	166644340.774	0.000
Kainic acid	0.249	1.015	0.811	0.030
3-Isopropenylpentanedioic acid	0.203	1.014	1.267	0.021
Glycerylphosphorylethanolamine	0.000	1.012	7.798	0.000
Quadrone	0.282	1.011	0.855	0.038
19-hydroxytelocinobufagin	0.266	1.009	0.814	0.034
5'-Hydroxypiroxicam	0.000	1.008	0.640	0.000
4-Methoxycinnamic acid	0.015	1.007	1.486	0.001
5,8-tetradecadienoic acid	0.000	1.006	20.451	0.000
Fenleuton	0.278	1.005	1.099	0.037
PtdIns-(3,4)-P2 (1,2-dihexanoyl)	0.109	1.004	1.982	0.008
4-Oxooctanoylcarnitine	0.044	1.001	0.678	0.002
2-Methylbenzoic acid	0.184	1.001	1.209	0.018

[1]Control, PFDG-15% and PFDG-30% represent the group without PEDG supplementation, the group with 15% PFDG substituting 15% concentrate, and the group with 30% PFDG substituting 30% concentrate, respectively (*n*=6).

[2]Q-value was used to adjust the false discovery rate in the comparisons.

[3]VIP=variable importance in the projection.

[4]FC=Fold change，which expressed as the ratio of average metabolite expression PFDG-15% group to Control group. For up-regulated metabolites, the folding change translates to a corresponding value greater than 1.

Table S2 Information on differential metabolites in the Control group vs. PFDG-30% group[1].

Differential Metabolites	Q-value[2]	VIP[3]	FC[4]	*P*
9,10-Epoxystearic acid	0.026	25.242	2.338	0.003
12-Hydroxystearic acid	0.007	19.555	2.838	0.000
Methyl 14-methyl-8-hexadecenoate	0.120	12.991	1.634	0.022
Hydroxyzine	0.017	12.451	1.707	0.001
Petroselinic acid	0.042	12.189	2.079	0.005
PA(8:0/i-18:0)	0.004	11.294	0.596	0.000
S-Japonin	0.098	10.729	1.954	0.016
Octadecanedioic acid	0.001	10.627	5.334	0.000
5S-HETE-d8	0.099	10.208	1.536	0.016
Schisanhenol	0.078	9.343	1.578	0.012
7-Methyl-2-(2-furyl)-1,8-naphthyridine-4(1H)-one	0.006	8.331	1.909	0.000
2-Methylhexadecanoic acid	0.055	8.043	2.134	0.007
4α-formyl-4β-methyl-5α-cholesta-8,24-dien-3β-ol	0.000	7.688	0.010	0.000
C16 Sphinganine	0.074	7.394	1.320	0.011
3E,13Z-Octadecadienal	0.037	7.205	1.976	0.004
4-(D-Glucopyranosyloxy)-4,4'-diaponeurosporene	0.006	6.955	0.404	0.000
Dodecanedioic acid	0.135	6.868	0.712	0.026
2-(5,8-Tetradecadienyl)cyclobutanone	0.074	6.526	1.846	0.011
(2s)-2-[[(2s)-2-Acetamido-5-[[n-(Methylcarbamoyl)carbamimidoyl]amino]pentanoyl]-Methyl-Amino]-3-Phenyl-Propanoic Acid	0.028	6.504	1.766	0.003
9S,11R,15S-trihydroxy-2,3-dinor-13E-prostaenoic acid-cyclo[8S,12R]	0.011	6.306	1.555	0.001
8,11,14,17-eicosatetraenoic acid	0.026	6.267	1.532	0.003
12(S),20-DiHETE	0.000	6.052	6.346	0.000
Cycluron	0.000	6.035	61.664	0.000
Pexacerfont	0.180	6.035	1.135	0.041
9,10-Epoxy-18-hydroxy-octadecanoic acid	0.028	5.805	1.911	0.003
Barbital	0.000	5.786	5.291	0.000
Saccharin	0.020	5.583	0.681	0.002
1-(2-methoxy-17Z-tetracosenyl)-sn-glycero-3-phosphoserine	0.003	5.157	0.526	0.000
Dibutyl decanedioate	0.080	5.080	1.807	0.012
Coronin	0.026	5.048	7.309	0.003
Hydantoin-5-propionic acid	0.020	4.958	0.762	0.002
Avenoleic acid	0.061	4.925	1.672	0.008
Ricinoleic acid	0.042	4.925	2.095	0.005
Pennelliiside C	0.001	4.902	22.610	0.000
Berkeleylactone F	0.001	4.861	1.941	0.000
Sebacic acid	0.025	4.846	0.902	0.002
2-[[(1R)-2-[Bis(carboxymethyl)amino]cyclohexyl]-[(2S)-2-[bis(carboxymethyl)amino]-3-(4-isothiocyanatophenyl)propyl]amino]acetic acid	0.001	4.816	2.306	0.000
Uridine 5'-monophosphate	0.036	4.816	0.687	0.004
Scopolin	0.001	4.779	2.202	0.000

PS(22:0/0:0)	0.006	4.761	0.621	0.000
Guanosine monophosphate	0.153	4.742	0.620	0.032
Glyzarin	0.205	4.636	0.918	0.050
Finerenone	0.106	4.635	1.610	0.018
Disodium phosphate	0.024	4.564	1.719	0.002
N-arachidonoyl isoleucine	0.160	4.537	2.371	0.034
N-(15-methyl-3-(13-methyl-4Z-tetradecenoyloxy)-hexadecanoyl)-glycine methyl ester	0.087	4.472	1.630	0.013
PC(16:0/18:2(9E,11E))	0.018	4.405	2.440	0.002
DG(20:3(8Z,11Z,14Z)-2OH(5,6)/0:0/a-15:0)	0.053	4.389	9.164	0.007
12S-hydroxy-16-heptadecynoic acid	0.010	4.377	1.823	0.001
Diethylene glycol dimethacrylate	0.001	4.271	0.650	0.000
17-Octadecynoic acid	0.121	4.261	1.160	0.022
Cohibin C	0.025	4.216	23.169	0.002
Metharbital	0.000	4.170	2.915	0.000
Benzofuran, 4,7-dimethyl-	0.000	4.017	2.685	0.000
Phenol A	0.002	3.994	0.593	0.000
Hydroxy Tyrosol -Acetate	0.002	3.988	0.086	0.000
6-Hydroxypentadecanedioic acid	0.000	3.873	20.160	0.000
Gly-arg-gly-asp-ser	0.000	3.835	6.130	0.000
Adenosine monophosphate	0.033	3.820	0.637	0.004
4,4-Dimethylcholesta-8,14,24-trienol	0.000	3.817	0.067	0.000
PE(P-16:0/15:0)	0.019	3.782	1.577	0.002
ent-16-F1t-PhytoP	0.041	3.777	0.710	0.005
PPA(16:0/18:1(9Z))	0.016	3.775	6.159	0.001
Eugenosedin-A	0.018	3.685	2.388	0.002
Retrorsine	0.029	3.673	1.467	0.003
Theonellasterol E	0.028	3.669	2.479	0.003
Enterodiol glucuronide	0.079	3.627	0.801	0.012
3,6,9,12-Tetraazatetradecanedioic acid, 3,6,9,12-tetrakis(carboxymethyl)-	0.000	3.602	6.295	0.000
1-((4-Methylsulfonyl)phenyl)-3-trifluoromethyl-5-(4-fluorophenyl)pyrazole	0.121	3.599	0.405	0.022
4-(4-Hydroxyphenyl)-2-butanone O-[2,6-digalloylglucoside]	0.000	3.572	6.261	0.000
11-dehydro-TXB2-d4	0.180	3.541	1.270	0.041
Glunicate	0.108	3.535	0.909	0.019
Sordarin	0.000	3.532	2.958	0.000
Tetraethylene glycol	0.202	3.522	1.334	0.049
[Bala8]-Neurokinin A(4-10)	0.029	3.483	2.936	0.003
4-Amino-5-methylamino-2',7'-difluorescein	0.018	3.453	0.698	0.002
4-amino-6,7-dihydro-5H-cyclopenta[b]pyridin-2-yl 4-methylbenzenesulfonate	0.000	3.428	6.386	0.000
Mupirocin	0.106	3.373	0.461	0.018
2,6-Diamino-4-hydroxy-5-N-methylformamidopyrimidine	0.000	3.352	15.447	0.000
CDP-DG(i-21:0/i-24:0)	0.085	3.306	0.684	0.013
PG(14:0/0:0)	0.052	3.252	1.930	0.007
5-Nitroxystearic acid	0.016	3.231	18.501	0.001

Spermidine	0.133	3.179	0.526	0.026
ascr#24	0.000	3.172	21.453	0.000
Cyclokirilodiol	0.191	3.166	1.799	0.045
Punicic acid	0.099	3.124	1.144	0.016
Cer(d16:1/TXB2)	0.099	3.118	0.692	0.016
Acenocoumarol	0.110	3.108	1.318	0.019
Sodium palmitate	0.006	3.102	1.527	0.000
Aloeresin B	0.026	3.092	0.791	0.003
10-methoxy-octadecanoic acid	0.084	3.077	2.470	0.013
1-O-(2R-hydroxy-pentadecyl)-sn-glycerol	0.076	3.049	1.276	0.011
Haematopodin	0.009	3.046	1.983	0.001
N2-Maltulosylarginine	0.053	3.005	1.561	0.007
Pantheric Acid A	0.171	2.993	1.377	0.038
Heptaethylene glycol monododecyl ether	0.098	2.990	1.196	0.016
2,6-Diamino-9-(2-hydroxyethoxymethyl)purine	0.057	2.984	0.905	0.007
gamma-Glutamylproline	0.001	2.978	2.822	0.000
Amrubicinol (mixture)	0.081	2.974	1.859	0.012
Hexadecanedioic acid	0.002	2.967	3.338	0.000
Sodium octadecanoate	0.089	2.959	1.361	0.014
Cer(d16:1/LTE4)	0.014	2.957	2.520	0.001
Indoleacrylic acid	0.101	2.948	0.590	0.017
Atenolol	0.000	2.948	15.360	0.000
5Z-Dodecenoic acid	0.019	2.929	0.720	0.002
DG(17:1(9Z)/18:0/0:0)[iso2]	0.055	2.920	1.480	0.007
Flazine	0.000	2.902	21.629	0.000
Posaconazole	0.011	2.900	0.652	0.001
Sarcoehrendin D	0.005	2.892	5.160	0.000
PG(14:1(9Z)/0:0)	0.148	2.851	1.522	0.030
8,11-Eicosadiynoic acid	0.024	2.842	1.574	0.002
Mycinonic acid III	0.012	2.824	0.801	0.001
3-oxo-tetracosanoic acid	0.000	2.820	35.207	0.000
Ecabet	0.040	2.809	1.801	0.005
PA(a-21:0/10:0)	0.104	2.791	64.099	0.018
OSU03012	0.024	2.729	1.776	0.002
4-(4-Fluorobenzyl)-2-p-tolyl-1,2,4-thiadiazolidine-3,5-dione	0.103	2.727	0.400	0.017
Morph	0.000	2.726	2.842	0.000
(+)-Isopilocarpine	0.000	2.726	13.938	0.000
Pilocarpine	0.044	2.711	0.489	0.005
Amantadine	0.006	2.692	3.079	0.000
1-(6-[3]-ladderane-hexanoyl)-2-(8-[3]-ladderane-octanyl)-sn-glycero-3-phospho-(1'-sn-glycerol)	0.011	2.664	25.553	0.001
Biocytin	0.022	2.645	1.722	0.002
Isomugineic acid	0.000	2.642	0.565	0.000
N-Benzoylaspartic acid	0.000	2.632	8.265	0.000

Sodium phosphate (Na(H2PO4))	0.050	2.630	1.539	0.006
(2R)-2-Amino-6-[[(3S,4R,5R)-3,4,5,6-tetrahydroxy-2-oxohexyl]amino]hexanoic acid	0.001	2.621	0.699	0.000
Hydroxyprolylhydroxyproline	0.003	2.611	2.645	0.000
N-Oleoyl Glycine	0.025	2.609	1.255	0.002
Ascorbyl palmitate	0.001	2.567	6.387	0.000
Arginyl-prolyl-proline	0.005	2.550	1.969	0.000
Phaseolic acid	0.005	2.549	0.553	0.000
(4E,6Z)-3-Hydroxydeca-4,6-dienoylcarnitine	0.161	2.539	2.673	0.034
Euchrenone b3	0.097	2.506	1.549	0.016
Procurcumadiol	0.011	2.506	0.595	0.001
Duloxetine	0.102	2.503	0.585	0.017
4-Hydroxy-3-methoxy-2,10-bisaboladien-9-one	0.000	2.499	1.959	0.000
Infectocaryone	0.000	2.486	19.939	0.000
6-Hexyltetrahydro-2H-pyran-2-one	0.110	2.475	0.730	0.019
Carboxyfluorescein diacetate	0.080	2.455	1.542	0.012
Suberic acid	0.162	2.452	0.787	0.035
(±)-(E)-13-Hydroxy-10-oxo-11-octadecenoic acid	0.183	2.447	0.619	0.042
Indolepyruvate	0.000	2.421	28.282	0.000
L-Monomenthyl glutarate	0.007	2.419	0.803	0.000
PA(13:0/15:1(9Z))	0.002	2.416	0.476	0.000
N-Succinyl-L,L-2,6-diaminopimelate	0.000	2.409	9.105	0.000
3-Hydroxy-2-oxoindole	0.000	2.408	7.373	0.000
Manassantin B	0.017	2.407	0.356	0.001
4-Hydroxyphenylretinamide	0.029	2.376	1.723	0.003
Leu-Pro-Ile	0.078	2.367	1.757	0.012
PPA(18:1(9Z)/18:1(9Z))	0.009	2.366	33.085	0.001
Glucocorticoid receptor agonist	0.029	2.342	1.638	0.003
oscr#23	0.000	2.341	79.630	0.000
(-)-Malyngolide	0.033	2.337	1.536	0.004
N-Acetylproline	0.003	2.323	1.762	0.000
Pimelic acid	0.002	2.304	0.688	0.000
15R-PGD2	0.051	2.300	1.481	0.006
Tyrosyl-Leucine	0.003	2.296	0.603	0.000
(1S)-1-(3-Methoxyphenyl)-2-[4-[4-[(propan-2-ylideneamino)oxymethyl]phenyl]triazol-1-yl]ethanol	0.112	2.289	2.069	0.020
8-HETE	0.055	2.275	2.189	0.007
Telbivudine	0.001	2.271	2.583	0.000
Tumonoic Acid A	0.005	2.254	3.668	0.000
1,3-Dihydro-(2H)-indol-2-one	0.108	2.254	0.515	0.019
Ergosine	0.003	2.214	6.294	0.000
L-Glutamic acid, N-((((1S)-1-carboxy-5-((((4-iodophenyl)amino)carbonyl)amino)pentyl)amino)carbonyl)-	0.118	2.207	0.283	0.021

3-[2-[(2R)-2-Phenyl-4-[(4-pyridin-4-ylphenyl)methyl]morpholin-2-yl]ethyl]-1H-quinazoline-2,4-dione	0.003	2.204	2.162	0.000
Glycyl-l-histidyl-l-lysine	0.000	2.169	2.929	0.000
PS(P-20:0/21:0)	0.003	2.165	0.537	0.000
Methoxybrassenin A	0.180	2.164	1.108	0.041
Iloperidone	0.000	2.159	6.002	0.000
11-Hydroxyyangonin	0.000	2.158	0.779	0.000
dihydroxyphaseic acid	0.045	2.150	0.577	0.005
5'-Deoxy-5'-fluorouridine	0.000	2.148	17.758	0.000
Anguvetin	0.018	2.139	2.356	0.002
Ethyl 3-((6-(4,5-dihydro-1H-benzo[d]azepin-3(2H)-yl)-2-(pyridin-2-yl)pyrimidin-4-yl)amino)propanoate	0.042	2.138	2.015	0.005
(S)-N-(4,5-Dihydro-1-methyl-4-oxo-1H-imidazol-2-yl)alanine	0.000	2.119	3.835	0.000
N-(1-Hydroxyethyl)octadecanamide	0.008	2.119	2.777	0.001
Tarenninoside A	0.016	2.118	0.666	0.001
N-Lauroyl Threonine	0.005	2.110	1.767	0.000
(9S,12S,16R)-d14-10-PhytoF[10S,13S]	0.042	2.103	0.801	0.005
4-(4-Fluorophenyl)-2-(4-hydroxyphenyl)-5-(4-pyridyl)imidazole	0.102	2.093	0.613	0.017
dCMP	0.116	2.092	0.767	0.021
Topostin D654	0.037	2.063	0.565	0.004
Normorphine 3-glucuronide	0.089	2.050	0.669	0.014
Ethyl 4-decenoate	0.185	2.020	0.815	0.042
Tambulin 3,5-diacetate	0.156	2.018	1.231	0.033
PGD2-d4	0.082	2.018	1.250	0.012
Stercobilinogen	0.061	2.013	4.302	0.008
MG(PGD1/0:0/0:0)	0.037	2.010	0.665	0.004
N-Acetyltryptamine	0.122	2.008	0.271	0.022
5-alpha-Cholestane-3-beta,5,6-beta-triol, 6-acetate 3-(hydrogen succinate)	0.177	2.007	0.476	0.040
Abscisic alcohol	0.001	1.997	0.743	0.000
HMBOA hexose	0.161	1.990	1.848	0.034
Isoleucyl-Valine	0.030	1.987	0.494	0.003
Triamcinolone	0.003	1.979	3.586	0.000
Momorcharaside B	0.114	1.969	0.352	0.020
Polyglycerol esters of fatty acids	0.000	1.965	433.708	0.000
Acacetin 7-(4'''-acetylrutinoside)	0.073	1.963	1.461	0.010
12-hydroxy-10-dodecenoic acid	0.023	1.956	0.681	0.002
Toxin T2 tetrol	0.000	1.952	4.996	0.000
Penicillin V	0.001	1.946	0.580	0.000
Furo(3,4-b)pyridine-3-carboxylic acid, 1,4,5,7-tetrahydro-4-(2-(difluoromethoxy)phenyl)-2-methyl-5-oxo-, ethyl ester	0.079	1.940	0.621	0.012
23-Hydroxybetulinic acid	0.085	1.937	1.653	0.013
Ibudilast	0.047	1.936	0.312	0.006

Cer(d16:1/PGJ2)	0.008	1.935	0.603	0.001
3-(4-Hydroxyphenyl)-3,5,6,8-tetrahydro-2H-chromene-4,7-dione	0.180	1.921	1.203	0.041
Catechin 7,3'-di-O-gallate	0.094	1.914	0.305	0.015
gamma-Glutamylhydroxyproline	0.017	1.912	0.775	0.001
(+)-cis-abscisic aldehyde	0.000	1.907	2.895	0.000
2,6,6-Trimethyl-1-cyclohexen-1-acetaldehyde	0.126	1.906	0.725	0.024
Petroformynic acid B	0.008	1.905	8.182	0.001
HARMOL	0.007	1.889	1.864	0.000
3'-Hydroxyamobarbital	0.001	1.883	2.658	0.000
Piroxantrone	0.000	1.882	9.493	0.000
1alpha,25-dihydroxy-24-oxo-23-azavitamin D2 / 1alpha,25-dihydroxy-24-oxo-23-azaergocalciferol	0.002	1.866	0.375	0.000
TRIOXIFENE	0.004	1.864	2.696	0.000
2',6'-Dihydroxy-4'-methoxy-3'-(2-hydroxybenzyl)dihydrochalcone	0.040	1.851	1.727	0.005
Latrunculin A	0.165	1.849	1.638	0.036
(2s)-2-[6-(Sulfooxy)naphthalen-2-yl]propanoic acid	0.000	1.838	0.543	0.000
LysoPA(22:5(7Z,10Z,13Z,16Z,19Z)/0:0)	0.185	1.835	0.561	0.042
11-nitro-1-undecene	0.041	1.832	1.189	0.005
Dec-5-enedioylcarnitine	0.003	1.831	0.540	0.000
Riddelliine	0.000	1.828	19.498	0.000
Proflavine	0.004	1.816	2.104	0.000
Aldosine	0.000	1.812	16.027	0.000
2-Hydroxy-4-[(1R)-1-hydroxy-8-methyl-6-oxononyl]-3-methyl-2H-furan-5-one	0.002	1.809	0.708	0.000
Hydroxyprolyl-Glutamine	0.013	1.801	2.542	0.001
19-Hydroxyprostaglandin E2	0.074	1.798	0.784	0.011
Coumarin 102	0.000	1.794	5.389	0.000
N-(4-Amino-5-methoxy-2-methylphenyl)benzamide	0.000	1.789	178.931	0.000
6,7-Epoxy-3Z,9Z-tricosadiene	0.071	1.777	2.713	0.010
5(6)-Epoxy Prostaglandin E1	0.002	1.772	0.695	0.000
Malvalic acid	0.126	1.767	1.884	0.023
PE-NMe2(18:3(6Z,9Z,12Z)/14:0)	0.004	1.767	16.561	0.000
Traumatic acid	0.080	1.764	0.726	0.012
Isovitexin 7-O-(6'''-caffeoyl)-beta-D-glucopyranoside	0.012	1.763	0.667	0.001
Hexylresorcinol	0.161	1.760	0.899	0.034
4-Oxo-1-(3-pyridyl)-1-butanone	0.000	1.756	5.990	0.000
3Z,6Z,9Z-Heptadecatriene	0.113	1.753	1.873	0.020
Beta-Tyrosine	0.015	1.748	0.423	0.001
6-hydroxyoct-4-enoylglycine	0.000	1.747	9.601	0.000
19-Hydroxy-PGE2	0.109	1.747	0.726	0.019
9-Fluorocortisone	0.060	1.744	1.225	0.008
12-oxo-PDA	0.008	1.738	1.401	0.000
glycodeoxycholic acid	0.084	1.733	1.828	0.013
4-amino-4-deoxychorismate	0.000	1.729	3.412	0.000

Isoleucylproline	0.000	1.727	6.205	0.000
Cyclopentolate	0.017	1.725	0.599	0.001
Butyrolactone derivative	0.001	1.725	2.789	0.000
2,6-Di-tert-butyl-1,4-benzenediol	0.090	1.723	0.792	0.014
Alanylhydroxyproline	0.000	1.711	2.565	0.000
Methyl-3H-thymidine	0.000	1.700	3.853	0.000
Apetalolide	0.175	1.699	1.406	0.039
22:3(5Z,9Z,19Z)(13Me,17Me,21Me)	0.119	1.698	0.828	0.022
Histidinyl-Lysine	0.001	1.696	9.287	0.000
Coriandrinonediol	0.046	1.692	1.923	0.006
2,6-Diaminopurine 2',3'-dideoxyriboside	0.039	1.690	0.800	0.004
Neamine	0.000	1.686	742811104.363	0.000
9-hydroxy-traumatin	0.004	1.681	0.722	0.000
2-Acetylaminophenoxazin-3-one	0.000	1.679	2.596	0.000
Undecanedioic acid	0.102	1.678	0.857	0.017
Indigo Carmine	0.000	1.673	5.134	0.000
Caffeinol	0.000	1.671	49.173	0.000
Ulexone D	0.092	1.666	1.237	0.015
Stearoyllactic acid	0.005	1.665	22.516	0.000
Ethylenediaminetetraaceticacid	0.114	1.655	0.928	0.020
Calcitriol	0.000	1.654	40.137	0.000
thioinosine monophosphate	0.001	1.654	0.537	0.000
(2E,8E)-10-hydroxydeca-2,8-dien-4-ynoylcarnitine	0.000	1.653	5.513	0.000
Met-Gln-Cys-Asn-Ser	0.002	1.652	4.413	0.000
D-Xylonate	0.000	1.648	2.203	0.000
1-O-(2R-methoxy-4Z-docosenyl)-sn-glycerol	0.001	1.645	3.770	0.000
N-palmitoyl threonine	0.005	1.630	3.811	0.000
((2-Amino-3-((2-amino-3-((carboxymethyl)amino)-3-oxopropyl)dithio)propanoyl)amino)acetic acid	0.001	1.629	0.570	0.000
PS(O-16:0/0:0)	0.026	1.626	0.551	0.003
Manoalide	0.001	1.620	7.098	0.000
Isopentenyladenine-9-N-glucoside	0.019	1.619	0.680	0.002
(Z)-15-Oxo-11-eicosenoic acid	0.007	1.617	7.811	0.000
Atevirdine	0.066	1.614	1.381	0.009
Prolylproline	0.005	1.612	1.656	0.000
Glutamine-glutamate	0.087	1.609	0.742	0.014
2beta-Hydroxytestosterone	0.008	1.607	0.674	0.001
N-(3-hydroxy-decanoyl)-homoserine lactone	0.001	1.606	0.493	0.000
2,3-Bis(4-hydroxyphenyl)propionitrile	0.000	1.605	5.146	0.000
Oxibendazole	0.101	1.604	0.824	0.017
sterculate	0.138	1.600	1.912	0.027
1-Hydroxy-6-methoxypyrene	0.019	1.600	0.753	0.002

Tetradecanoylcarnitine	0.006	1.599	46.626	0.000
DG(10:0/0:0/8:0)	0.016	1.597	3.326	0.001
1alpha,25-dihydroxy-23-azavitamin D3 / 1alpha,25-dihydroxy-23-azacholecalciferol	0.153	1.595	2.418	0.032
Talaromycin A	0.039	1.590	0.667	0.004
2-Phenylaminoadenosine	0.000	1.588	4.776	0.000
Anhydrocinnzeylanine	0.156	1.579	1.898	0.033
Petromylidene A	0.139	1.578	1.680	0.027
4-Hydroxy-4-(3-pyridyl)-butanoic acid	0.000	1.577	1.855	0.000
(2S)-7-hydroxy-5-methoxy-6-methylflavanone	0.091	1.575	0.894	0.015
Tamsulosin	0.001	1.573	3.226	0.000
3-amino-2-naphthoic acid	0.001	1.572	1.299	0.000
1-Hydroxyvitamin D5	0.045	1.571	0.437	0.005
PIP3(18:2(9Z,12Z)/20:1(11Z))	0.004	1.569	8.784	0.000
Isoflupredone acetate	0.091	1.565	1.793	0.015
Iprovalicarb	0.034	1.564	0.339	0.004
N-oleoyl glutamine	0.000	1.563	5.157	0.000
Cerulenin	0.000	1.562	8.328	0.000
9,10,18-TriHOME(12Z)	0.125	1.562	0.746	0.023
3,10-dihydroxydecanoic acid	0.018	1.550	0.692	0.002
Tyrosine methylester	0.043	1.547	0.696	0.005
1-O-Pentylglycerol	0.164	1.540	1.240	0.035
Olaparib	0.127	1.538	0.362	0.024
DG(20:4(5Z,7E,11Z,14Z)-OH(9)/0:0/i-12:0)	0.171	1.532	0.702	0.038
Isoleucyl-Glutamate	0.098	1.526	0.702	0.016
3-Ethylheptadecan-2-one	0.007	1.524	0.613	0.000
Galaxolide	0.150	1.523	1.139	0.031
3-Hydroxyquinolin-2(1h)-One	0.186	1.522	0.499	0.043
2,6,10,10-Tetramethyl-1-oxaspiro[4.5]decan-6-ol	0.031	1.522	1.662	0.003
3,11,12-Trihydroxy-1(10)-spirovetiven-2-one	0.005	1.521	0.718	0.000
12-HETE	0.164	1.520	1.583	0.035
Acetamide, 2-(diethylamino)-N-(2-(octyloxy)phenyl)-	0.000	1.517	7.379	0.000
Cyclocalopin D	0.000	1.517	4.620	0.000
dolichyl beta-D-glucosyl phosphate	0.100	1.516	0.559	0.017
N-[3-(13-methyl-tetradecanoyloxy)-13-methyl-hexadecanoyl] glycyl serine	0.041	1.508	0.496	0.005
FS4 toxin	0.053	1.505	0.780	0.007
Ganoderol B	0.070	1.504	1.685	0.010
5,7,3'-Trihydroxy-6,4',5'-trimethoxyflavanone	0.029	1.504	0.723	0.003
19-hydroxytelocinobufagin	0.000	1.502	0.565	0.000
5'-Amino-5'-deoxyadenosine	0.006	1.500	0.542	0.000
1-(2-Hydroxyethoxy)methyl-5-methyluracil	0.000	1.497	8.273	0.000
Soyasapogenol C	0.094	1.494	0.073	0.015
Isolimocitrol 3-beta-D-glucoside	0.041	1.492	0.714	0.005
GalCer(d18:0/26:1)	0.047	1.491	2.272	0.006

PYRROLIZIDINE	0.142	1.490	0.515	0.028
Rupintrivir	0.171	1.488	2.240	0.038
(5x,6x)-5,6-Epoxyergosta-7,22-dien-3-ol	0.031	1.481	2.751	0.003
N-[4-[(6-Methoxypyrimidin-4-yl)sulfamoyl]phenyl]acetamide	0.000	1.481	3.539	0.000
(24E)-15alpha-Acetoxy-3alpha-hydroxy-23-oxo-7,9(11),24-lanostatrien-26-oic acid	0.128	1.480	0.690	0.024
Gibberellin A53	0.005	1.472	1.323	0.000
Dibenzylamine	0.018	1.465	2.566	0.002
Non-sulfonylurea	0.000	1.463	4.090	0.000
Glycylproline	0.026	1.461	1.605	0.003
Cerevisterol	0.003	1.461	2.187	0.000
(4alpha,5alpha)-4,14-Dimethyl-9,19-cyclocholest-20-en-3-one	0.194	1.461	1.475	0.046
PC(18:2(10E,12Z)+=O(9)/17:0)	0.003	1.459	1.509	0.000
Formylfusarochromanone	0.000	1.458	4.667	0.000
Gentiatibetine	0.000	1.458	3.648	0.000
4-p-Coumaroyl-1,5-quinolactone	0.016	1.457	0.730	0.001
Glycyllysylarginine	0.006	1.455	1.517	0.000
2-Methylhippuric acid	0.000	1.454	0.147	0.000
7-Ethyl-4-tridecen-6-one	0.019	1.452	1.617	0.002
Phenethylamine glucuronide	0.000	1.451	4.042	0.000
20-Hydroxy-leukotriene B4	0.062	1.450	1.861	0.008
3-(Aminomethyl)-2,5,9-trimethyl-7H-furo[3,2-g]chromen-7-one	0.000	1.448	57.757	0.000
L-DOPA n-Butyl Ester	0.000	1.448	9.310	0.000
2-[[5-[3-(Dimethylamino)propyl]-2-methylpyridin-3-yl]amino]-9-(trifluoromethyl)-5,7-dihydropyrimido[5,4-d][1]benzazepine-6-thione	0.007	1.445	2.099	0.000
9E-tetradecenoic acid	0.025	1.438	1.779	0.002
2-Cyanohept-2-enoic acid	0.000	1.433	8.010	0.000
6-Hydroxyhexadecanedioylcarnitine	0.093	1.433	0.620	0.015
Potassium phosphate (K(H2PO4))	0.079	1.433	1.502	0.012
6-[Ethyl-(3-isobutoxy-4-isopropylphenyl)amino]nicotinic acid	0.138	1.431	1.330	0.027
S-(2-Methylbutanoyl)-dihydrolipoamide	0.002	1.427	0.716	0.000
Leucylphenylalanine	0.066	1.426	0.661	0.009
4-Bis(2-hydroxyethyl)amino-L-phenylalanine	0.000	1.425	4.178	0.000
N-Desmethyllevomepromazine	0.001	1.418	0.697	0.000
3-Indolebutyric acid	0.001	1.413	0.147	0.000
Catechin 5,4'-di-O-beta-D-glucopyranoside	0.000	1.410	6.811	0.000
Oxidized glutathione	0.122	1.408	1.161	0.023
Droxidopa	0.095	1.407	1.446	0.015
Carazolol	0.000	1.404	7.099	0.000
Mefloquine	0.067	1.404	0.482	0.009
Hydrocortisone cypionate	0.035	1.401	0.620	0.004
3S-hydroxy-4R-methyl-2S-(n-eicos-11?-yn-19?-enyl)butanolide	0.017	1.400	22.822	0.001
Pranlukast	0.115	1.396	1.452	0.020
1-(8-[5]-ladderane-octanyl)-2-(8-[3]-ladderane-octanyl)-sn-glycero-3-phospho-(	0.129	1.396	0.658	0.025

1'-sn-glycerol)				
(4S,6R)-6-[(1E)-4,4-Bis(4-fluorophenyl)-3-(1-methyltetrazol-5-yl)buta-1,3-dien yl]-4-hydroxyoxan-2-one	0.000	1.395	11.124	0.000
Glucoiberin	0.018	1.394	0.578	0.002
Risbitin	0.003	1.391	0.717	0.000
3-Azetidinecarboxylic acid	0.009	1.388	1.758	0.001
Psilocybine	0.056	1.388	2.313	0.007
3Z-Tetradecen-1-ol	0.165	1.387	1.309	0.036
Corey PG-Lactone Diol	0.001	1.386	0.760	0.000
Dehydroxymethylflazine	0.000	1.385	30.104	0.000
3-(8,11,14-Pentadecatrienyl)phenol	0.131	1.384	0.288	0.025
Porphobilinogen	0.004	1.383	4.530	0.000
2-Methyl-3-(2-methylphenyl)pyrido[2,3-d]pyrimidin-4-one	0.094	1.382	2.467	0.015
Fexinidazole	0.116	1.380	0.868	0.021
Tripropyl phosphate	0.000	1.375	2.106	0.000
3,5-Pyridinedicarboxylic acid, 1,4-dihydro-2,4,6-trimethyl-, diethyl ester	0.000	1.375	75.726	0.000
(2R,7"S)-8-(1-phenyl-2-carboxyethyl)pinocembrin	0.020	1.374	2.507	0.002
7-hydroxy-dodecanoic acid	0.000	1.373	334.253	0.000
1,4,10,13-Tetraoxa-7,16-diazacyclooctadecane	0.010	1.368	0.626	0.001
(1R,6R)-6-hydroxy-2-succinylcyclohexa-2,4-diene-1-carboxylate	0.001	1.367	0.277	0.000
26,26,26-trifluoro-25-hydroxy-27-norvitamin D3	0.000	1.366	0.504	0.000
2-(5-Methoxy-1H-indol-3-yl)ethyl acetate	0.000	1.366	4.219	0.000
10E-Pentadecen-6,8-diynoic acid	0.003	1.366	0.715	0.000
Diacetolol	0.119	1.365	0.684	0.022
Metoprolol acid	0.000	1.361	30.221	0.000
Nervonyl carnitine	0.060	1.360	0.590	0.008
6beta-Hydroxycampest-4-ene-3-one	0.154	1.360	0.698	0.032
Fistulosin	0.206	1.359	2.214	0.050
Glutamylisoleucine	0.031	1.358	0.712	0.003
(+)-(R)-2-(2,4-Difluorophenyl)-1-(3-((E)-4-(2,2,3,3-tetrafluoropropoxy)styryl)-1,2,4-triazol-1-yl)-3-(1,2,4-triazol-1-yl)propan-2-ol	0.079	1.354	0.835	0.012
Mirabegron	0.168	1.352	0.767	0.037
DG(10:0/0:0/10:0)	0.003	1.350	542529063.155	0.000
19(20)-EpDPE	0.032	1.349	17.942	0.003
5'-Phosphoribosyl-N-formylglycinamide	0.000	1.348	32.353	0.000
Norketobemidone	0.000	1.348	2.967	0.000
Ac-Pro-Gly-Pro-OH	0.142	1.346	0.608	0.028
Frenolicin B	0.095	1.343	0.836	0.015
Deuteroporphyrin IX	0.109	1.341	0.361	0.019
trans-4-Aminocyclohexanecarboxylic acid	0.087	1.339	1.229	0.014
cis-3-Hexenyl lactate	0.027	1.338	0.744	0.003

4,1-Benzoxazepine	0.064	1.337	0.479	0.009
Methylene bisacrylamide	0.000	1.335	3.338	0.000
PGF1a alcohol	0.002	1.333	20.750	0.000
[6]-Dehydrogingerdione	0.009	1.327	0.706	0.001
1-(6-Hydroxy-2-azabicyclo[2.2.1]heptane-3-carbonyl)pyrrolidine-2-carbonitrile	0.186	1.326	1.120	0.043
5,7-Dihydroxy-8,4'-dimethoxyisoflavone	0.159	1.324	1.360	0.034
Gorgostane-3beta,5alpha,6beta,11alpha,12beta-pentol 12-acetate	0.006	1.324	0.434	0.000
Cer(d18:2(4E,14Z)/20:5(7Z,9Z,11E,13E,17Z)-3OH(5,6,15))	0.009	1.322	0.357	0.001
Pretyrosine	0.000	1.322	0.462	0.000
N'-Hydroxysaxitoxin	0.115	1.317	0.707	0.020
Albendazole	0.040	1.313	0.800	0.005
N-(3-Aminopropyl)-4-methyl-2-nitrobenzenamine	0.013	1.312	1.297	0.001
DIACETYLDIDEISOVALERYL-RHODOMYRTOXIN	0.195	1.311	0.896	0.046
(22E,24R)-15alpha-hydroxyergosta-4,6,8(14),22-tetraen-3-one	0.006	1.309	16.951	0.000
5-Phosphoribosylamine	0.092	1.307	0.502	0.015
valine lactate	0.000	1.305	8.866	0.000
1-(3-Furanyl)-6,7-dihydroxy-4,8-dimethyl-1-nonanone	0.009	1.305	0.751	0.001
D-Fucose	0.012	1.303	20.425	0.001
Fucose 1-phosphate	0.000	1.303	0.251	0.000
Persenone B	0.007	1.301	37.112	0.000
1alpha-hydroxy-18-(4-hydroxy-4-methyl-2-pentynyloxy)-23,24,25,26,27-penta norvitamin D3 / 1alpha-hydroxy-18-(4-hydroxy-4-methyl-2-pentynyloxy)-23,24,25,26,27-penta norcholecalciferol	0.204	1.300	2.328	0.049
(10S,11S)-Pterosin C	0.000	1.297	3.374	0.000
Artabsinolide D	0.002	1.295	0.704	0.000
Sagequinone methide A	0.135	1.294	0.706	0.026
Phenylbutyrylglutamine	0.052	1.291	0.564	0.007
Syringetin 3-rhamnoside	0.200	1.290	1.107	0.048
Cer(d16:2(4E,6E)/22:0)	0.131	1.289	1.817	0.025
PA(P-16:0/17:1(9Z))	0.101	1.285	1.561	0.017
Arginyl-glycyl-aspartic acid	0.019	1.281	0.676	0.002
(9E)-9-nitrooctadecenoic Acid	0.044	1.279	1.913	0.005
3-Methoxyanthranilate	0.000	1.276	3.522	0.000
(2R,3Z)-Phycocyanobilin	0.138	1.269	1.640	0.027
Ketobemidone	0.000	1.264	30.552	0.000
Kynurenic acid	0.040	1.259	0.524	0.005
5,8-tetradecadienoic acid	0.000	1.256	55.024	0.000
Indirubin-3'-monoxime	0.006	1.256	2.208	0.000
22S-hydroxy-24-methylene-cholesterol	0.029	1.254	1.579	0.003
8,20-DiHETE	0.002	1.252	0.667	0.000
Quadrone	0.003	1.249	0.735	0.000
Cer(d18:2(4E,14Z)/PGJ2)	0.115	1.247	0.532	0.020
Arborinine	0.076	1.244	2.860	0.011

2-Butylbenzothiazole	0.003	1.242	0.768	0.000
Pyranomammea B	0.039	1.240	2.938	0.004
2alpha-Hydroxyalantolactone	0.007	1.240	0.727	0.000
5-Hydroxyenterolactone	0.069	1.240	0.539	0.010
Thr-Leu	0.000	1.239	11.495	0.000
1-(beta-D-Ribofuranosyl)-1,4-dihydronicotinamide	0.001	1.239	7.968	0.000
OH-Diaponeurosporene glucoside ester	0.105	1.239	0.390	0.018
Nitroglycerin	0.003	1.237	0.647	0.000
PA(15:1(9Z)/0:0)	0.065	1.232	1.379	0.009
Calycanthidine	0.009	1.227	0.431	0.001
N-Hexadecanoylpyrrolidine	0.017	1.224	1.599	0.001
Fumonisin FP3	0.003	1.223	73.776	0.000
(23S)-1alpha,23-dihydroxy-25,26-didehydrovitamin D3 / (23S)-1alpha,23-dihydroxy-25,26-didehydrocholecalciferol	0.006	1.223	11.078	0.000
3alpha-Hydroxy-5beta-chola-7,9(11)-dien-24-oic Acid	0.205	1.220	0.555	0.049
MG(0:0/20:5(5Z,8Z,10E,14Z,17Z)-OH(12)/0:0)	0.011	1.220	0.580	0.001
Radiprodil	0.017	1.219	0.697	0.001
4,11,13,15-Tetrahydroridentin B	0.009	1.214	0.783	0.001
Vomifoliol	0.042	1.204	0.764	0.005
beta-D-ribosylnicotinate	0.000	1.202	3.820	0.000
Siduron	0.083	1.201	0.706	0.013
[5-[2-[4-(4-Benzhydrylpiperazin-1-ium-1-yl)phenyl]ethoxycarbonyl]-2,6-dimethyl-4-(3-nitrophenyl)-4H-pyridin-3-ylidene]-methoxymethanolate	0.115	1.197	0.600	0.020
7-Methylinosine	0.000	1.196	19.044	0.000
Invertin	0.133	1.195	3.000	0.026
Naloxonazine	0.067	1.193	9.632	0.009
Dyphylline	0.000	1.184	30.505	0.000
L-Norleucine	0.003	1.178	1.586	0.000
Triethylhexanoin	0.020	1.178	13.466	0.002
Metiazinic acid	0.000	1.177	44.959	0.000
Nonoxynol-9	0.084	1.173	0.735	0.013
12-Oxo-2,3-dinor-10,15-phytodienoic acid	0.001	1.172	1.735	0.000
Cis-stilbene oxide	0.002	1.172	2.489	0.000
3,4-dimethyl-5-carboxyethyl-2-furanbutanoic acid	0.007	1.170	0.724	0.000
Leucyl-Gamma-glutamate	0.001	1.168	0.594	0.000
Cyclopassifloside X	0.023	1.167	2.678	0.002
3-(2-Furanylmethyl)-1H-pyrrole	0.116	1.167	0.333	0.021
LysoPA(18:0/0:0)	0.157	1.160	1.135	0.033
Carminomycin II	0.196	1.157	1.165	0.046
5-O-Methylleridol	0.000	1.155	2.634	0.000
Isolubimin	0.001	1.154	1.422	0.000
Proline glutamate	0.000	1.151	3.436	0.000
Arabsin	0.002	1.151	0.623	0.000
Proline betaine	0.012	1.150	1.348	0.001

Eriotriochin	0.174	1.147	1.646	0.039
(S)-[10]-Gingerol	0.000	1.143	27.442	0.000
4-Hydroxyquinoline	0.020	1.141	0.513	0.002
6,2'4'-Trihydroxy-2-phenylbenzofuran	0.000	1.137	43.633	0.000
N1-Acetylspermidine	0.035	1.136	0.593	0.004
Pomalidomide	0.000	1.135	6.412	0.000
PA(O-20:0/0:0)	0.119	1.132	1.208	0.022
Potassium dodecanoate	0.001	1.130	1.410	0.000
5Z,8Z,11Z,14Z-Eicosatetraenedioic acid	0.174	1.130	0.566	0.038
(2-Acetyloxy-3-hydroxypropyl) (E)-octadec-9-enoate	0.001	1.130	0.594	0.000
Tyrosyl-Asparagine	0.015	1.128	0.497	0.001
Tizoxanide glucuronide	0.172	1.123	0.651	0.038
Sodium citrate	0.004	1.122	0.670	0.000
L-cis-Cyclo(aspartylphenylalanyl)	0.059	1.118	0.575	0.008
Methoxyfenozide	0.134	1.118	0.658	0.026
Buibuilactone	0.070	1.114	0.752	0.010
Pyroglutamylvaline	0.061	1.111	0.625	0.008
N-((1,2,3,5,6,7-Hexahydro-s-indacen-4-yl)carbamoyl)-4-(2-hydroxypropan-2-yl)furan-2-sulfonamide	0.022	1.111	0.540	0.002
N-Palmitoyl Cysteine	0.119	1.108	5.346	0.022
Blennin D	0.006	1.107	0.734	0.000
Antofloxacin	0.074	1.107	2.055	0.011
4,6-Pentacosanedione	0.084	1.106	2.447	0.013
2-Acetamido-2,6-dideoxygalactose	0.040	1.105	1.203	0.005
(2xi,6xi)-7-Methyl-3-methylene-1,2,6,7-octanetetrol	0.168	1.103	0.780	0.037
5-Hydroxyheptanoylcarnitine	0.000	1.103	0.378	0.000
Polypropylene glycol (m w 1,200-3,000)	0.080	1.103	1.367	0.012
Lysylleucine	0.011	1.102	0.529	0.001
6-Methoxymellein	0.000	1.102	4.565	0.000
Carnosol	0.009	1.101	1.415	0.001
alatolide	0.008	1.100	0.763	0.001
Carboxydextran	0.050	1.100	1.540	0.006
N-alpha-Acetyl-L-citrulline	0.021	1.100	0.683	0.002
13-Heptadecyn-1-ol	0.170	1.099	1.049	0.037
Glycyl-Phenylalanine	0.066	1.097	0.602	0.009
Furoparadine	0.006	1.096	2.307	0.000
9-Pentadecenoic acid	0.018	1.096	1.636	0.002
KB 2	0.001	1.095	20.833	0.000
(Z)-Resveratrol	0.000	1.094	0.769	0.000
Ibandronate	0.066	1.092	2.740	0.009
Serylvaline	0.004	1.091	0.567	0.000
Borapetoside	0.028	1.088	0.435	0.003
L-gamma-Glutamyl-beta-phenyl-beta-L-alanine	0.026	1.087	0.628	0.003
5-((Z)-nonadec-8-en-1-yl)resorcinol	0.040	1.086	0.502	0.005

8-Methoxy-6,7-methylenedioxycoumarin	0.000	1.086	4.556	0.000
O-Desmethylangolensin	0.084	1.084	0.341	0.013
2-Acetyl-6-methylpyridine	0.000	1.082	17.159	0.000
Pamapimod	0.024	1.081	0.752	0.002
5-Hydroxydecanedioylcarnitine	0.001	1.081	0.522	0.000
Metomidate	0.049	1.079	2.182	0.006
Serylphenylalanine	0.010	1.077	1.442	0.001
ortho-Hydroxyphenylacetic acid	0.003	1.077	1.645	0.000
Alitame	0.004	1.073	0.619	0.000
MG(18:1(9Z)-O(12,13)/0:0/0:0)	0.127	1.072	1.833	0.024
(Z)-Tamarindienal	0.000	1.072	1.568	0.000
4,5-Dihydroniveusin A	0.040	1.072	0.804	0.005
Cappariloside B	0.075	1.072	1.170	0.011
Diethyl 1,4-dihydro-2,6-dimethyl-3,5-pyridinedicarboxylate	0.000	1.072	5.511	0.000
Hymenoxon	0.006	1.070	0.760	0.000
Malonylglycine	0.116	1.069	0.799	0.021
3-[(2-Oxoacetyl)oxy]-4-(trimethylazaniumyl)butanoate	0.013	1.068	4.647	0.001
Glutamylleucine	0.051	1.067	0.813	0.006
D-Arginine	0.050	1.066	0.576	0.006
DG(10:0/20:4(6Z,8E,10E,14Z)-2OH(5S,12R)/0:0)	0.129	1.063	0.544	0.025
[(2R,5S,15S)-2,15-Dimethyl-14-oxotetracyclo[8.7.0.0^{2,7}.0^{11,15}]heptadec-7-en-5-yl]oxidanesulfonic acid	0.000	1.062	16.861	0.000
2,3-Dinor-TXB2	0.029	1.061	0.744	0.003
Glutaminylisoleucine	0.013	1.061	0.618	0.001
4-(2-Naphthyloxy)-2-butyne-1-amine	0.000	1.060	207.729	0.000
1-Hydroxy-2,2,5,5-tetramethyl-2,5-dihydro-1h-pyrrole-3-carboxamide	0.039	1.060	1.213	0.004
Olomoucine	0.000	1.057	227.934	0.000
6-Methyladenine	0.042	1.056	0.685	0.005
Leucyl-leucine	0.156	1.055	0.540	0.033
Z-Ala-ONp	0.020	1.054	0.670	0.002
Fostemsavir	0.125	1.053	0.345	0.023
2-Hydroxy-22-methyltetracosanoic acid	0.038	1.052	1.609	0.004
11R-HETE	0.020	1.052	1.839	0.002
NAD	0.100	1.052	1.437	0.017
Isamoxole	0.000	1.048	289712119.693	0.000
Droperidol	0.008	1.048	0.811	0.000
1,2,3,4-Tetrahydro-b-carboline-1,3-dicarboxylic acid	0.133	1.048	1.675	0.026
Norbadione A	0.179	1.046	0.451	0.040
Beta-Alanyl-CoA	0.117	1.044	1.470	0.021
3,3'-Sulfonyldianiline	0.111	1.044	0.655	0.019

Thromboxane B2	0.121	1.043	0.505	0.022
8alpha-Hydroxy-gama-tocopherone	0.194	1.043	1.274	0.046
xymedon	0.000	1.042	8.969	0.000
Brassicanal A	0.002	1.041	0.583	0.000
3-Methyloxindole	0.103	1.040	0.539	0.017
b-D-Glucuronopyranosyl-(1->3)-a-D-galacturonopyranosyl-(1->2)-L-rhamnose	0.000	1.038	8.264	0.000
3'-N'-Acetylfusarochromanone	0.064	1.038	1.441	0.009
Niridazole	0.001	1.036	0.741	0.000
(+)-gamma-Hydroxy-L-homoarginine	0.000	1.034	0.000	0.000
tyrosine lactate	0.003	1.033	1.673	0.000
L-Carnitine	0.046	1.033	1.537	0.006
2-Hydroxyquinoline-3-carboxylic acid	0.020	1.032	1.179	0.002
2-Methyl-4-pentenoic acid	0.137	1.031	0.650	0.027
13,14-dihydro-15-keto-PGE1	0.004	1.031	7.765	0.000
Tolmetin	0.006	1.030	0.045	0.000
Oxprenolol	0.059	1.029	1.223	0.008
6'-Sialyllactosamine	0.015	1.029	2.192	0.001
L-Thyronine	0.000	1.028	28.839	0.000
Nipradilol	0.000	1.028	0.292	0.000
Isokobusone	0.038	1.027	0.747	0.004
Artemisin	0.080	1.026	0.757	0.012
Quadrangolin A; 2-[(1S,2S,4aR,8aS)-1-Hydroxy-4a-methyl-8-methylenedecahydro-2-naphthalenyl]acrylic acid	0.003	1.025	0.779	0.000
Aegelinol	0.149	1.025	1.149	0.031
PG(14:1(9Z)/12:0)	0.004	1.021	0.339	0.000
L-Agaridoxin	0.007	1.021	0.587	0.000
Ibuprofen	0.003	1.020	0.815	0.000
(2R,3S)-3-Hydroxy-1,1-dimethylpyrrolidin-1-ium-2-carboxylate	0.036	1.020	1.553	0.004
Diphenylhydantoic acid	0.000	1.019	5.376	0.000
N-[(2Z)-2-Methoxyiminopropyl]-7H-purin-6-amine	0.001	1.018	0.345	0.000
(8R,9R,10S,13S,14S)-13-Methyl-1,4,5,6,7,8,9,10,11,12,14,15,16,17-tetradecahydrocyclopenta[a]phenanthrene	0.107	1.018	1.181	0.018
Campesteryl glucoside	0.010	1.017	0.546	0.001
Nicametate	0.000	1.017	6.661	0.000
Epiheterodendrin	0.001	1.017	8.666	0.000
Pseudoyohimbine	0.007	1.015	0.569	0.000
Elastin	0.019	1.015	0.739	0.002
PtdIns-(3,4)-P2 (1,2-dihexanoyl)	0.008	1.012	2.771	0.001
Icaritin 3-rhamnoside	0.000	1.012	2.726	0.000
Amafolone	0.112	1.010	0.386	0.020
Ethyl vanillin	0.005	1.010	0.705	0.000
Mevalonic acid	0.001	1.007	2.784	0.000
7-Hydroxyoctadecanoic acid	0.006	1.004	4.731	0.000

Lidofenin	0.015	1.004	0.598	0.001
4alpha-Hydroxymethyl-4beta-methyl-5alpha-cholesta-8,24-dien-3beta-ol	0.001	1.001	0.015	0.000

[1]Control, PFDG-15% and PFDG-30% represent the group without PEDG supplementation, the group with 15% PFDG substituting 15% concentrate, and the group with 30% PFDG substituting 30% concentrate, respectively (n=6).

[2]Q-value was used to adjust the false discovery rate in the comparisons.

[3]VIP=variable importance in the projection.

[4]FC=Fold change, which expressed as the ratio of average metabolite expression PFDG-30% group to Control group. For up-regulated metabolites, the folding change translates to a corresponding value greater than 1.
